# Nudge theories and strategies influencing adult health behaviors and outcomes in COPD management: a systematic review

**DOI:** 10.3389/fpubh.2024.1404590

**Published:** 2024-11-05

**Authors:** Qiuhui Wu, Ruobin Zhang, Li Tao, Wenting Cai, Xinrui Cao, Zhi Mao, Jinping Zhang

**Affiliations:** ^1^Department of Pharmacy, Nanjing Drum Tower Hospital, Affiliated Hospital of Medical School, Nanjing University, Nanjing, China; ^2^Department of Pharmacy, Nanjing Drum Tower Hospital, School of Basic Medicine and Clinical Pharmacy, China Pharmaceutical University, Nanjing, China; ^3^Department of Pharmacy, College of Medicine, Yangzhou University, Yangzhou, China; ^4^Department of Pharmacy, Tumor Hospital of Gansu Province, Lanzhou, China; ^5^Department of Critical Care Medicine, The First Medical Center, Chinese PLA General Hospital, Beijing, China

**Keywords:** chronic obstructive pulmonary disease, health behavior, self-management, nudge, systematic review

## Abstract

**Objective:**

Chronic obstructive pulmonary disease (COPD) is a chronic respiratory disease with high prevalence and mortality, and self-management is a key component for better outcomes of COPD. Recently, nudging has shown promising potential in COPD management. In the present study, we conducted a systematic review to collate the list of nudges and identified the variables that influence nudging.

**Methods:**

We undertook a systematic review. We employed database searches and snowballing. Data from selected studies were extracted. The risk of bias was assessed using the Cochrane Effective Practice and Organization of Care risk of bias tool. The study is registered with PROSPERO, CRD42023427051.

**Results:**

We retrieved 4,022 studies from database searches and 38 studies were included. By snowballing, 5 additional studies were obtained. Nudges were classified into four types: social influence, gamification, reminder, and feedback. Medication adherence, inhalation technique, physical activity, smoking cessation, vaccination administration, exercise capacity, self-efficacy, pulmonary function, clinical symptoms, and quality of life were analyzed as targeted health behaviors and outcomes. We found medication adherence was significantly improved by reminders via mobile applications or text materials, as well as feedback based on devices. Additionally, reminders through text materials greatly enhance inhalation techniques and vaccination in patients.

**Conclusion:**

This review demonstrates nudging can improve the health behaviors of patients with COPD and shows great potential for certain outcomes, particularly medication adherence, inhalation techniques, and vaccination. Additionally, the delivery modes, the patient characteristics, and the durations and seasons of interventions may influence the successful nudge-based intervention.

**Clinical trial registration:**

This review has been registered in the international Prospective Registry of Systematic Evaluation (PROSPERO) database (identifier number CRD42023427051).

## 1 Introduction

Chronic obstructive pulmonary disease (COPD) is a prevalent and severe chronic respiratory ailment that is characterized by persistent and typically progressive airflow obstruction, leading to an irreversible decline in pulmonary function (1). Globally, COPD has become a prominent public health concern, imposing a considerable burden of mortality and morbidity (2). According to the statistics from the World Health Organization, COPD is regarded as the third most prevalent cause of death internationally (3). Due to its chronic and progressive condition, COPD significantly impacts the lifestyle and quality of life of patients. To enhance health outcomes, quality of life, and long-term prognosis of patients, effective COPD care encompasses a range of behavioral interventions, pharmacological therapies, respiratory therapies, exercise rehabilitation, smoking cessation, and vaccination (1, 4, 5). In addition to professional guidance, the management of COPD necessitates patients’ autonomous involvement by implementing effective and sustainable behavioral modifications to attain enduring disease control (6–8).

The new term “nudge,” originally derived from behavioral economics, has recently gained popular attention as an innovative approach for encouraging patients with chronic illnesses to modify their health-related behaviors (9). Nudge was first introduced and defined by economist Thaler and his associate Sunstein, which means the process of assisting individuals in making better judgments on a predictable routine when they are faced with choices, without prohibiting any options or drastically altering the incentives that drive the behaviors (10). In contrast to commands or persuasion, nudging does not restrict patients’ freedom of choice (10). It seeks to modify the choice architecture or the environment in which decisions are made (10). Examples of this include rearranging food items to promote healthier eating or laying warning images on cigarette packs to encourage quitting smoking (11, 12). There is evidence supporting the effectiveness of nudges in improving health behaviors among patients with chronic diseases such as heart disease and COPD (13, 14).

Currently, scholars cannot come to a consensus on the definition of nudge yet, and different approaches have been taken to classify nudges, such as the MINDSPACE classification proposed by Dolan et al. (15) and Munscher et al.’s (16) classification by intervention design. Consequently, we are not sure of exactly what type of nudge works effectively to improve the health behaviors and outcomes of patients with COPD. On the other hand, the context of decision-making influences nudges (17). For example, patients with varying characteristics may react differently to the same nudge, and the same nudge may have conflicting effects when delivered in different modes (17–19). In order to implement effective nudges, these contexts also require additional investigation.

The article aims to perform a systematic review that thoroughly assesses the applications of nudge theory and strategies in connection with health behaviors and outcomes among patients with COPD. This review helps to further understand the potential influence of the characteristics on the effectiveness of nudge interventions by presenting scientific supporting data on the use of nudge in the management of COPD. Overall, it provides novel evidence-based practice for researchers, politicians, and medical practitioners to implement nudges for COPD patients.

## 2 Methods

This review has been registered in the international Prospective Registry of Systematic Evaluation (PROSPERO) database (identifier number CRD42023427051). Registration was open until June 1, 2023.

### 2.1 Nudge theory and strategies

We referred to the interpretation and classification of nudges by Meske et al. (20) and Munscher et al. (16) to guide us to include the following strategies as the subcategories of nudges in this review.

#### 2.1.1 Social influence

Social influence can be categorized into two basic types. The first type involves information. When a large number of individuals act or think in a certain way, knowledge about what might be beneficial in terms of actions or thoughts is disseminated through their collective behavior and thinking patterns. The second type is peer pressure. In this case, one may choose to conform to societal norms or follow the majority due to concerns about how others, such as family members, perceive them, aiming to gain approval or avoid disapproval (11).

#### 2.1.2 Gamification

Gamification refers to the use of game design elements in non-game contexts, to motivate and engage through fun and enjoyment in a game setting (21).

#### 2.1.3 Reminder

Reminders can make a big difference since they ensure that people can act promptly at the right moment and avoid procrastinating being distracted by other duties or becoming inert (12).

#### 2.1.4 Feedback

Feedback makes own behavior visible. Giving feedback on one’s behavior removes many of the attentional and mental barriers that prevent people from accessing this knowledge in their daily lives. Devices and tools that offer feedback include pedometers that count steps and smart electricity meters displaying energy consumption (16).

### 2.2 Information sources and search strategy

We employed a two-arm search strategy including database searches and snowballing. We searched PubMed, Embase, Web of Science, Cochrane Library, Scopus, PsycINFO, and EconLit for relevant articles. We carefully defined the terms and keywords and determined the search strategy (see Supplementary Table 1). The search filter was set to English only. The search was performed on June 26, 2023. The references of included studies were also manually checked for appropriate sources. Deduplicates were removed using the reference management software EndNote 20, followed by a meticulous manual deduplication process.

### 2.3 Inclusion and exclusion criteria

Eligible studies were selected based on specific inclusion and exclusion criteria. The inclusion criteria included adults diagnosed with COPD; interventions based on the theory of behavioral nudges; study designs that featured an appropriate control, such as randomized controlled trials or pre-post studies, where the control group received non-nudges like usual care; reported outcomes that encompassed medication adherence, inhalation technique, physical activity, smoking cessation, vaccine behavior, self-efficacy in health behaviors, as well as pulmonary function, clinical symptoms, and quality of life as health outcomes; and the literature search was limited to studies published in English. Exclusion criteria were as follows: patients with Acute Exacerbation of Chronic Obstructive Pulmonary Disease (AECOPD), conference abstracts, letters, comments, case reports, case series, preclinical studies, review articles, descriptive studies such as case–control studies and cohort studies, other non-relevant studies, and studies with no relevant results. Table 1 lists the PICOS for this systematic review.

**Table 1:** PICOS for this systematic review.

Elements	Contents
P (Population)	Adults diagnosed with COPD
I (Intervention)	Interventions based on the theory of behavioral nudges
C (Comparison)	Non-nudged interventions, like usual care
O (Outcome)	Health behaviors and outcomes such as medication adherence, inhalation technique, physical activity, smoking cessation, vaccine behavior, self-efficacy, pulmonary function, clinical symptoms, and quality of life
S (Study design)	An appropriate control, such as randomized control or pre-post control

### 2.4 Study selection

The entire screening process was conducted by two independent reviewers (W-QH and Z-RB). Any disagreements were resolved through discussion. If necessary, a third reviewer (ZM) performed assistance and reached a consensus with all investigators. Based on the inclusion and exclusion criteria, a two-round screening process was implemented. Initially, screening involved reading the titles and abstracts, followed by a comprehensive examination of the full texts.

### 2.5 Data extraction

The data was extracted independently by two review authors using a pre-designed data extraction form. Information extracted included publication information (e.g., title, first author, year of publication, study design, country), patient characteristics (e.g., mean age, sex, sample size), intervention or control characteristics (e.g., nudge intervention and control description, duration of intervention) and targeted behaviors and health outcomes, and limitations.

### 2.6 Quality assessment

The risk of bias in each study was assessed using the Cochrane Effective Practice and Organization of Care risk of bias tool (22), which has nine standard criteria for the study with a separate control group and seven standard criteria for the interpreted time series study. Each study was independently assessed by two reviewers against each of the criteria assessing a score of either low risk, high risk, or unclear risk of bias according to the Cochrane Effective Practice and Organization of Care risk of bias tool (22).

### 2.7 Data synthesis

Due to the heterogeneity of interventions, outcome measurements, and study designs in the included studies, it would not have been possible to provide substantial results by performing a meta-analysis. Instead, to confirm the effective context of nudge interventions, we reported statistical significance based on the type of nudge and included outcome measures, and used a vote counting approach for narrative synthesis.

## 3 Results

### 3.1 Study selection

**Figure 1:**
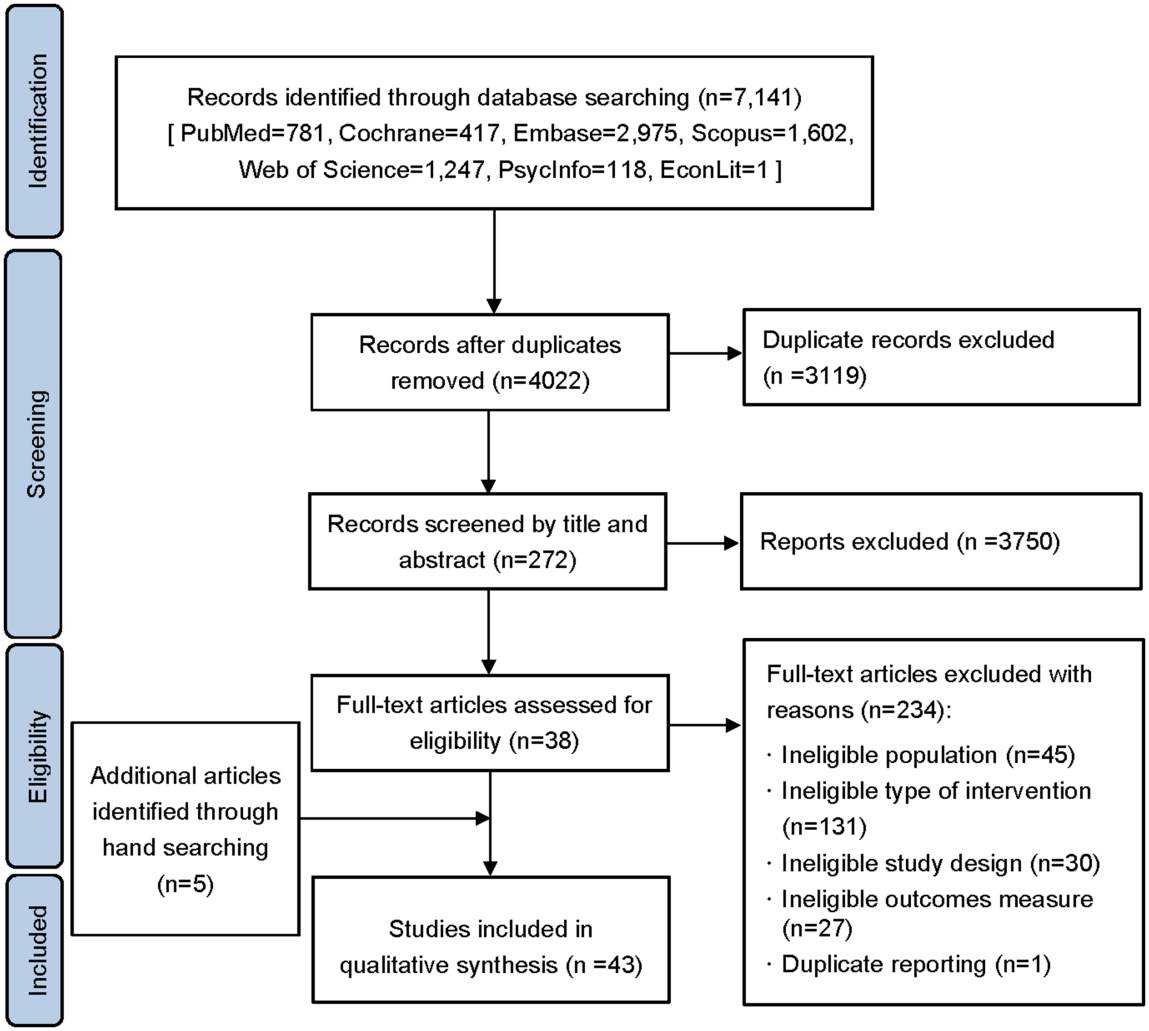
Flow chart of the literature screening process.

A total of 4,022 articles were identified in the initial search from the database after the exclusion of 3,119 duplicates. After screening the titles and abstracts, 3,750 articles were excluded because they did not meet the inclusion criteria. 272 articles in total were selected for full-text screening. After that, 234 articles were disqualified (Figure 1). Five additional articles were added after manual searching. Finally, 43 studies were included in the review (Figure 1). The features of the study were shown in Table 2. Among them, three studies were lacking in the information on gender and one study only indicated age range without mean age. The sample sizes in most studies were less than 50. Thirty studies were randomized controlled studies, and ten studies were performed in the United States. Supplementary Tables 2, 3 provide specifics regarding the study’s characteristics and nudge strategies.

**Table 2:** Characteristics of studies.

Population selection	Number of studies
Mean age (years)	
[40,50)	2
[50,60)	2
[60,70)	29
≥70	9
Age provided as a range	1
Male (%)	
<30	1
[30,60)	15
[60,90)	19
≥90	5
Gender not reported	3
Sample size	
<50	21
[50,100)	7
[100,200)	10
≥200	5
Country/region	
North America	
USA	10
Europe	
UK	4
Other European countries ^a^	17
Asia-Pacific countries ^b^	12
Study design	
RCT	31
Non-RCT	12

a Other European countries include Germany, France, Switzerland, Portugal, Netherlands, Norway, Italy, Poland, Spain and Turkey.

b Asia-Pacific countries include Australia, Chile, China, Indonesia, Vietnam, Korea and Japan.

### 3.2 Quality assessment

The results from the methodological risk of bias assessment of the included studies using the Cochrane Effective Practice and Organization of Care (EPOC) risk of bias tool are reported in Supplementary Table 4. Fifteen studies in all were classified as high risk: four for selection bias, six for dissimilar baseline outcomes or characteristics, five for high dropout rates leading to incomplete outcome data, and 3 for absence of blind assessment.

### 3.3 Characteristics of nudge interventions

As shown in Table 3, this study included a total of four different types of nudge interventions, including social influence (*n* = 5), gamification (*n* = 7), reminder (*n* = 6), and feedback (*n* = 25). Twenty-one of the 43 trials used multi-component interventions, meaning that in addition to nudging, they contained interventions including training, education, rehabilitation activities, general encouragement, and health outcome monitoring.

**Table 3:** Characteristics of nudge interventions.

Intervention	Social influence (*n* = 5)	Gamification (*n* = 7)	Reminder (*n* = 6)	Feedback (*n* = 25)
Multi-component	3	1	3	14
Single-component	2	6	3	11
Delivery mode				
Mobile applications	3	1	2	1
Web-based		1		
Pedometer/devices		5	1	24
Group sessions	2			
Text materials			3	
Targeted health behavior				
Physical activity	2	1		18
Exercise capacity	3	5	1	17
Smoking cessation				1
Influenza vaccination			1	
Medication adherence			3	3
Inhalation technique			1	
Self-efficacy	2		1	6
Pulmonary function		1	1	2
Clinical symptom		1		12
Quality of life	3	2	3	20
Duration of intervention				
<3 months	1	7	4	8
[3,6) months	3			8
[6,12) months			1	4
≥12 months	1		1	5

The most popular intervention was feedback nudges, and the most frequently used delivery modes (*n* = 24) were pedometers or other devices (23–46), along with mobile applications (47). Among the five studies employing social influence, two used group sessions (14, 48), and three utilized mobile applications (49–51) as the delivery modes. Of the seven gamification interventions, five involved devices such as consoles and headsets (52–56), one was web-based (57), and one used mobile applications (58). The interventions utilized various reminder devices, including wearable ones (59), while two others employed mobile applications (60, 61). Three other interventions employed text materials, including labels (62), cards (63), and written materials (64).

The nudge intervention of social influence primarily targeted exercise capacity, physical activity, and self-efficacy (14, 48–51). Exercise capacity and physical activity were the primary concerns of gamification (12, 52, 54–58). The main points of the reminder were self-efficacy, exercise capacity, vaccine behavior, inhalation technique, and medication adherence (59–64). The primary goals of the 25 studies that included feedback were self-efficacy, exercise capacity, and physical activity (23–47). Each kind of intervention looked into how it affected quality of life.

### 3.4 Characteristics of effective nudging measured

The results and health behavior data from the included studies are displayed in Table 4. Table 5 outlines the characteristics of effective nudge interventions. Variations in the targeted outcomes and method of delivering nudging interventions resulted in different intervention effects.

**Table 4:** Results of outcome measures of interest in the included studies.

First author (year)	Group (intervention/control)	*n*	Baseline measurement	Follow-up measurement	Within group significant result	Between groups significant result
Medication adherence						
Tashkin (1991) (24)	I	112	NA	The percent of patients actually used the inhaler two or more times daily according to the chronolog record: 78%	NA	*p* < 0.001
	C	85	NA	The percent of patients actually used the inhaler two or more times daily according to the chronolog record:52%	NA	
Simmons (1996) (43)	I	129	Not reported	Mean sets per day: Month 4: 1.93 ± 0.69 Month 8: 1.76 ± 0.83 Month 12: 1.74 ± 0.89 Month 16: 1.70 ± 0.89 Month 20: 1.56 ± 0.87 Month 24: 1.65 ± 0.89	Not reported	Month 4: *p* = 0.0035 Month 8: *p* = 0.0003 Month 12: *p* = 0.0007 Month 16: *p* = 0.0018 Month 20: *p* = 0.0190 Month 24: *p* = 0.0006
	C	102	Not reported	Mean sets per day: Month 4: 1.60 ± 0.83 Month 8: 1.31 ± 0.89 Month 12: 1.29 ± 0.91 Month 16: 1.27 ± 0.92 Month 20: 1.22 ± 0.97 Month 24: 1.16 ± 0.95	Not reported	
Song (2014) (64)	I	20	Medication adherence: 30.4 ± 4.8	Medication adherence: 33.0 ± 3.2	Not reported	*p* = 0.047
	C	20	Medication adherence: 31.6 ± 3.4	Medication adherence: 31.9 ± 4.8	Not reported	
Jolly (2018) (38)	I	Baseline: 273 Month 6: 219 Month12: 218	Medication adherence score: 1[0–2]^*^	Medication adherence score: Month 6: 1[0–2]^*^ Month 12: 1[0–1]^*^	Not reported	Month 6: *p* = 0.008
	C	Baseline: 265 Month 6: 255 Month12: 255	Medication adherence score: 1[0–2]^*^	Medication adherence score: Month 6: 1[0–2]^*^ Month 12: 1[0–2]^*^	Not reported	
Criner (2021) (61)	I	67	NA	The mean number of adherent sets of puffs/day: 1.61 ± 0.389	NA	*p* < 0.001
	C	70	NA	The mean number of adherent sets of puffs/day: 1.33 ± 0.509	NA	
Morfaw (2023) (60)	I	30	MMAS-8: 4.4 ± 1.591	MMAS-8: 6.4 ± 1.453	*p* < 0.05	NA
Inhalation technique						
Nguyen (2018) (62)	I	Baseline: 211 Month 1:163 Month 3:163 Month 6:139 Month12:102	Inhaler technique score: MDI:6.09 Turbuhaler®: 6.68	Inhaler technique score: MDI: Month 1 = 6.97; Month 3 = 7.49; Month 6 = 7.49; Month 12 = 6.93 Turbuhaler®: Month 1 = 7.24; Month 3 = 7.56; Month 6 = 7.56; Month 12 = 7.24	MDI: all points: *p* < 0.001 Turbuhaler®: Month 3: *p* = 0.001 Month 6: *p* < 0.001	NA
Smoking cessation						
Jolly (2018) (38)	I	Baseline: 289 Month 6: 267 Month12: 247	Smoking cessation rate: 26%	Smoking cessation rate: Month 6: 22% Month 12: 13%	Not reported	NS
	C	Baseline: 288 Month 6: 287 Month12: 226	Smoking cessation rate: 19%	Smoking cessation rate: Month 6: 18% Month 12: 25%	Not reported	
Influenza vaccination						
Vayisoglu (2019) (63)	I	44	NA	Influenza vaccination rate: 63.6%	NA	*p* = 0.001
	C	44	NA	Influenza vaccination rate: 29.5%	NA	
Physical activity						
de Blok (2006) (41)	I	8	Daily steps: 2082	Daily steps: 3512	Not reported	NS
	C	8	Daily steps: 2377	Daily steps: 2832	Not reported	
Wewel (2008) (23)	I	21	Activity per hour of monitoring (counts/h): 1061 ± 636	Activity per hour of monitoring (counts/h): 1330 ± 726	*p* = 0.007	NA
Hospes (2009) (32)	I	18	Daily steps: 7087 ± 4,058	Daily steps: 7872 ± 3,962	Not reported	*p* = 0.01
	C	17	Daily steps: 7539 ± 3,945	Daily steps: 6172 ± 3,194	Not reported	
Berry (2010) (14)	I	Baseline: 87 End: 61	Physical activity levels (kcals/week): Reported in diagram format	Physical activity levels (kcals/week): Reported in diagram format	Month 3: *p* = 0.004 Month 6: *p* = 0.005 Month12: *p* = 0.048	NS
	C	Baseline: 89 End: 69	Physical activity levels (kcals/week): Reported in diagram format	Physical activity levels (kcals/week): Reported in diagram format	Month 3: *p* = 0.002 Month 6: *p* = 0.039	
Moy (2012) (25)	I	24	Daily steps: 2908 ± 2,416	Daily steps: 4171 ± 2,970	*p* = 0.0054	NA
Cruz (2014) (34)	I	16	Daily steps (W1): 8638.23 ± 2408.14	Daily steps: W7: 10002.27 ± 2798.13 W12: 8858.43 ± 1641.80	W1 to W12: *p* = 0.026 W1 to W7: *p* = 0.050; W7 to W12: *p* = 0.048	NA
Tabak (2014) (47)	I	13	Daily steps: 5766 ± 965	Daily steps: 5603 ± 964	NS	Not reported
	C	16	Daily steps: 5256 ± 865	Daily steps: 4617 ± 865	NS	
Altenburg (2015) (39)	I	Baseline: 78 Month 3: 65 Month15: 50	Daily steps: 4292[2182–6,596]^*^	Daily steps: Changes after 3 months: 618[−137–1771]^*^ Changes after 15 months: 218[−1,423–1863]^*^	Not reported	Changes after 3 months: *p* = 0.001
	C	Baseline: 77 Month 3: 55 Month15: 51	Daily steps: 4132[2979–6,030]^*^	Daily steps: Changes after 3 months: −185[−1,425–969]^*^ Changes after 15 months: −201[1809–1,006]^*^	Not reported	
Kawagoshi (2015) (31)	I	12	The time spent walking (mins/day): Reported in diagram format	Changes after 12 months: 51.3 ± 63.7	*p* < 0.05	*p* = 0.036
	C	15	The time spent walking (mins/day): Reported in diagram format	Changes after 12 months: 12.3 ± 25.5	*p* < 0.05	
Mendoza (2015) (37)	I	Baseline: 52 End: 50	Daily steps: 4008 ± 2,253	Daily steps: Changes after 3 months: 3080 ± 3254.8	Not reported	*p* < 0.001
	C	Baseline: 50 End: 47	Daily steps: 3956 ± 2,723	Daily steps: Changes after 3 months: 138.3 ± 1950.4	Not reported	
Cruz (2016) (44)	I	13	Daily steps: 7161.5 ± 1708.1	Daily steps: Month 3: 10440.0 ± 4012.9 Month 6: 9747.9 ± 3511.8	*p* = 0.001	Month 3: *p* = 0.006 Month 6: *p* = 0.025
	C	13	Daily steps: 6617.1 ± 2914.2	Daily steps: Month 3: 6430.0 ± 2613.1 Month 6: 6481.3 ± 3454.4	*p* = 0.001	
Arbillaga-Etxarri (2018) (46)	I	132	Daily steps: 8069 ± 4,554	Daily steps: 8002 ± 4,635	NS	NS
	C	148	Daily steps: 7783 ± 3,847	Daily steps: 7825 ± 3,850	NS	
Burkow (2018) (51)	I	10	The average number of physical activity sessions per week (mean): 2.9	The average number of physical activity sessions per week (mean): 5.9	Not reported	Not reported
Jolly (2018) (38)	I	Baseline: 230 Month 6: 202 Month12: 191	Total MET minutes/week: 3242.2 ± 3284.2	Total MET minutes/week: Month 6: 3786.0 ± 3685.7 Month 12: 3214.3 ± 3578.4	Not reported	Month 6: *p* = 0.003
	C	Baseline: 236 Month 6: 237 Month12: 223	Total MET minutes/week: 3265.8 ± 3480.6	Total MET minutes/week: Month 6: 2920.6 ± 3195.0 Month 12: 2738.1 ± 3249.9	Not reported	
O’Neill (2018) (28)	I	Baseline:17 End: 14	Daily steps: 3305.6 ± 1960.2	Daily steps: 5332.0 ± 3070.7	Not reported	Not reported
	C	Baseline:23 End: 12	Daily steps: 3946.2 ± 2263.1	Daily steps: 4984.6 ± 3598.0	Not reported	
Kohlbrenner (2020) (35)	I	Baseline: 37 End: 29	Daily steps: 3708 ± 3,601	Daily steps: Changes after 3 months: 694 ± 1709 Changes after 12 months: −108 ± 1,057	Not reported	NS
	C	Baseline: 37 End: 31	Daily steps: 2451 ± 1819	Daily steps: Changes after 3 months: 423 ± 2,258 Changes after 12 months: −480 ± 1703	Not reported	
Park (2020) (33)	I	22	Daily steps: 5223.68 ± 2899.61	Daily steps: 6546.77 ± 2354.43	*p* < 0.05	NS
	C	20	Daily steps: 6756.26 ± 2978.77	Daily steps: 6890.39 ± 2967.73	NS	
Armstrong (2021) (27)	I	24	Daily steps: 3450 ± 2,168	Daily steps: 4426 ± 2,577	*p* = 0.001	*p* = 0.001
	C	24	Daily steps: 3446 ± 2,342	Daily steps: 3406 ± 2095	NS	
Geidl (2021) (45)	I	167	Daily steps: 5722.4 ± 2948.6	Daily steps: Week 6: 6875.0 ± 3229.5 Month 6: 6517.7 ± 3427.8	*p* < 0.05	NS
	C	160	Daily steps: 5934.5 ± 3101.0	Daily steps: Week 6: 6679.5 ± 3337.4 Month 6: 6234.0 ± 3357.6	*p* < 0.05	
Robinson (2021) (26)	I	75	Daily steps: 3176.6 ± 2211.6	Changes after 3 months: 645.95 ± 3394.6 Changes after 6 months: 672.90 ± 3399.0	Changes after 3/6 months: Not reported	Month 3: *p* = 0.005 Month 6: *p* < 0.001
	C	78	Daily steps: 3210.2 ± 2247.9	Changes after 3 months: −385.78 ± 3633.8 Changes after 6 months: −639.38 ± 3667.9	Changes after 3/6 months: Not reported	
Simmich (2021) (58)	I	9	Daily steps: 4730 ± 1959	Daily steps: 4649 ± 2,357	Not reported	Not reported
	C	9	Daily steps: 6394 ± 4,306	Daily steps: 5593 ± 4,277	Not reported	
Exercise capacity						
Giardino (2004) (29)	I	20	6MWD(m): 249 ± 97	6MWD(m): 432 ± 133	*p* < 0.01	NA
de Blok (2006) (41)	I	8	2MST: 36.6	2MST: 57.4	Not reported	NS
	C	8	2MST: 49.3	2MST: 55.1	Not reported	
Woo (2006) (48)	I	33	6MWD(m): 285 ± 96	6MWD(m): 303 ± 98	NS	NA
Wewel (2008) (23)	I	21	6MWD(m): 379.6 ± 115.3	6MWD(m): 411.4 ± 100.5	*p* = 0.030	NA
Hospes (2009) (32)	I	18	6MWD(m): 364.9 ± 45.1	6MWD(m): 387.4 ± 46.6	Not reported	NS
	C	17	6MWD(m): 351.4 ± 54.5	6MWD(m): 361.4 ± 66.6	Not reported	
Berry (2010) (14)	I	Baseline: 87 End: 61	6MWD(m): 410.7	6MWD(m): Month 3: 434.8 ± 8.8 Month 6: 426.7 ± 10.3 Month 12: 408.1 ± 10.5	Month 3: *p* < 0.05	NS
	C	Baseline: 89 End: 69	6MWD(m): 410.7	6MWD(m): Month 3: 428.7 ± 8.3 Month 6: 439.8 ± 9.9 Month 12: 430.5 ± 10.0	Month 3, 12: *p* < 0.05	
Cruz (2014) (34)	I	16	6MWD(m)(W1): 466.50 ± 81.56	6MWD(m): W12: 513.33 ± 86.18	W1 to W12: *p* = 0.001	NA
Song (2014) (64)	I	20	6MWD(m): 300.3 ± 86.6	6MWD(m): 333.5 ± 79.2	Not reported	NS
	C	20	6MWD(m): 290.0 ± 52.5	6MWD(m): 312.7 ± 72.1	Not reported	
Altenburg (2015) (39)	I	Baseline: 78 Month 3: 65 Month15: 50	6MWD(m): 454[361–509]^*^	6MWD(m): Changes after 3 months: 19.5[−5.6–45.2]^*^ Changes after 15 months: 22.8[2.4–51.2]^*^	Not reported	NS
	C	Baseline: 77 Month 3: 55 Month15: 51	6MWD(m): 450[351–530]^*^	6MWD(m): Changes after 3 months: 6.0[−18.5–40.6]^*^ Changes after 15 months: 11.2[−3.3–57.0]^*^	Not reported	
Kawagoshi (2015) (31)	I	12	6MWD(m): 369 ± 119	6MWD(m): 445 ± 138	*p* < 0.01	Not reported
	C	15	6MWD(m): 404 ± 148	6MWD(m): 467 ± 151	*p* < 0.01	
Mendoza (2015) (37)	I	Baseline: 52 End: 50	6MWD(m): 463.1 ± 83.2	6MWD(m): Changes after 3 months: 12.4 ± 34.6	Not reported	*p* = 0.03
	C	Baseline: 50 End: 47	6MWD(m): 469.7 ± 71.6	6MWD(m): Changes after 3 months: −0.7 ± 24.4	Not reported	
Cruz (2016) (44)	I	13	6MWD(m): 493.8 ± 63.0	6MWD(m): Month 3: 547.9 ± 47.9 Month 6: 540.4 ± 31.1	*p* < 0.001	NS
	C	13	6MWD(m): 476.2 ± 54.9	6MWD(m): Month 3: 529.7 ± 57.2 Month 6: 519.4 ± 50.8	*p* < 0.001	
Arbillaga-Etxarri (2018) (46)	I	132	6MWD(m): 499 ± 95	6MWD(m): 488 ± 106	*p* < 0.05	NS
	C	148	6MWD(m): 501 ± 83	6MWD(m): 493 ± 90	*p* < 0.05	
O’Neill (2018) (28)	I	Baseline:23 End: 16	ISWT (m): 253.0 ± 118.8	ISWT (m): 288.1 ± 107.0	Not reported	Not reported
	C	Baseline:26 End: 17	ISWT (m): 259.2 ± 140.6	ISWT (m): 280.0 ± 139.7	Not reported	
Wootton (2018) (30)	I	49	6MWD(m): 458 ± 87	6MWD(m): Changes after 14 months: −6 Changes from 2 months to 14 months: −23	Changes from 2 months to 14 months: *p* < 0.05	NS
	C	46	6MWD(m): 467 ± 80	6MWD(m): Changes after 14 months: −40 Changes from 2 months to 14 months: −39	Changes from 2 months to 14 months: *p* < 0.05	
Rutkowski (2019) (54)	I	34	6MWD(m): 469.9 ± 34.3	6MWD(m): 508.4 ± 44.3	*P* ≤ 0.05	NS
	C	34	6MWD(m): 494.9 ± 38.7	6MWD(m): 514.7 ± 33	*P* ≤ 0.05	
Sutanto (2019) (53)	I	10	6MWD(m): 376.6 ± 81.0	6MWD(m): 420 ± 77.6	*p* < 0.001	NS
	C	10	6MWD(m): 410.7 ± 105.3	6MWD(m): 477.5 ± 122.4	*p* < 0.001	
Kohlbrenner (2020) (35)	I	Baseline: 37 End: 29	1MSTS: 20.97 ± 7.04	1MSTS: Changes after 3 months: 0.74 ± 3.46 Changes after 12 months: 1.0 ± 7	Not reported	NS
	C	Baseline: 37 End: 31	1MSTS: 16.06 ± 8.72	1MSTS: Changes after 3 months: 1.81 ± 5.97 Changes after 12 months: −0.5 ± 6.9	Not reported	
Park (2020) (33)	I	22	6MWD(m): 378.32 ± 96.96	6MWD(m): 433.23 ± 107.23	*p* < 0.05	NS
	C	20	6MWD(m): 398.10 ± 78.67	6MWD(m): 437.60 ± 83.62	NS	
Rutkowski (2020) (55)	I/ET + VR	38	6MWD(m): 471.53	6MWD(m): 510.63	*p* = 0.000	ET vs. ET + VR: *p* = 0.011
	I/VR	34	6MWD(m): 487.91	6MWD(m): 523.38	*p* = 0.000	ET vs. VR: *p* = 0.031
	C/ET	34	6MWD(m): 492.07	6MWD(m): 508.3	*p* = 0.014	
Armstrong (2021) (27)	I	24	6MWD(m): 285 ± 92	6MWD(m): 339 ± 90	*p* = 0.001	NS
	C	24	6MWD(m): 276 ± 92	6MWD(m): 314 ± 99	*p* = 0.001	
Robinson (2021) (26)	I	75	6MWD(m): 360.8 ± 92.0	Changes after 3 months: 23.86 ± 82.97 Changes after 6 months: 25.14 ± 83.23	Changes after 3 months: Not reported Changes after 6 months: *p* = 0.010	NS
	C	78	6MWD(m): 357.2 ± 103.5	Changes after 3 months: 27.58 ± 83.99 Changes after 6 months: 37.41 ± 85.05	Changes after 3 months: Not reported Changes after 6 months: *p* < 0.001	
Rutkowski (2021) (56)	I	25	6MWD(MET): 6.12 ± 2.12	6MWD(MET): 6.75 ± 2.24	*p* < 0.0018	Not reported
	C	25	6MWD(MET): 5.98 ± 1.84	6MWD(MET): 6.76 ± 1.28	*p* < 0.0002	
Yao (2021) (50)	I	50	6MWD(m): 362.31 ± 91.24	6MWD(m): 423.67 ± 102.32	Not reported	*p* = 0.030
	C	50	6MWD(m): 364.57 ± 93.66	6MWD(m): 382.28 ± 84.95		
Colombo (2023) (57)	I	12	6MWD(m): 478.00 ± 80.44	6MWD(m): 520.50 ± 69.24	*p* < 0.05	NA
Norweg (2023) (36)	I	12	6MWD(m): 358.27 ± 87.85	6MWD(m): 397.64 ± 92.2	*p* = 0.01	Not reported
	C	4	6MWD(m): 322.94 ± 72.66	6MWD(m): 347.93 ± 84.71	Not reported	
Self-efficacy						
Giardino (2004) (29)	I	20	COPD self-efficacy: 49 ± 22	COPD self-efficacy: 62 ± 20	*p* < 0.01	NA
de Blok (2006) (41)	I	8	Self-efficacy: 25.3	Self-efficacy: 28.5	Not reported	NS
	C	8	Self-efficacy: 27.0	Self-efficacy: 26.6	Not reported	
Hospes (2009) (32)	I	18	LIVAS: 29.8 ± 7.9	LIVAS: 31.0 ± 8.9	Not reported	NS
	C	17	LIVAS: 28.4 ± 7.9	LIVAS: 28.5 ± 8.3	Not reported	
Cruz (2016) (44)	I	13	Self-efficacy Scale: 77.0 ± 12.0	Self-efficacy Scale: Month 3: 75.3 ± 12.7 Month 6: 79.5 ± 11.4	NS	NS
	C	13	Self-efficacy Scale: 82.4 ± 10.4	Self-efficacy Scale: Month 3: 85.7 ± 11.1 Month 6: 79.6 ± 13.0	NS	
Jolly (2018) (38)	I	Baseline: 287 Month 6: 247 Month12: 228	Stanford self efficacy scale: 8.3 ± 1.6	Stanford self efficacy scale: Month 6: 8.1 ± 1.7 Month 12: 8.1 ± 1.6	Not reported	NS
	C	Baseline: 284 Month 6: 275 Month12: 272	Stanford self efficacy scale: 8.0 ± 1.7	Stanford self efficacy scale: Month 6: 7.8 ± 1.8 Month 12: 7.7 ± 1.8	Not reported	
Vayisoglu (2019) (63)	I	44	SE Scale: Coping SE: 12.05 ± 1.43 Action SE: 10.86 ± 1.95	SE Scale: Coping SE: 13.11 ± 1.08 Action SE: 11.70 ± 2.01	Coping SE: *p* < 0.001 Action SE: *p* < 0.001	Coping SE: *p* < 0.001 Action SE: *p* < 0.001
	C	44	SE Scale: Coping SE: 11.39 ± 1.97 Action SE: 10.45 ± 2.52	SE Scale: Coping SE: 11.70 ± 2.01 Action SE: 10.93 ± 2.42	Action SE: *p* = 0.035	
Park (2020) (33)	I	22	SEMCD: 6.71 ± 1.93	SEMCD: 6.89 ± 1.75	NS	NS
	C	20	SEMCD: 6.47 ± 1.64	SEMCD: 6.69 ± 2.26	NS	
Yao (2020) (49)	I	64	ES-CA: 93.26 ± 11.23	ES-CA: 129.71 ± 23.22	Not reported	*p* < 0.05
	C	64	ES-CA: 94.13 ± 12.75	ES-CA: 109.25 ± 17.52	Not reported	
Yao (2021) (50)	I	50	health promotion self-care scale: 52.69 ± 9.36	health promotion self-care scale: 65.91 ± 11.39	Not reported	*p* < 0.001
	C	50	health promotion self-care scale: 52.74 ± 10.21	health promotion self-care scale: 56.36 ± 10.36		
Pulmonary function						
Esteve (1996) (42)	I	9	FEV1(% predicted): 33.2 ± 7.4	FEV1(% predicted): 40.1 ± 11.5	*p* = 0.038	NS
	C	10	FEV1(% predicted): 37.6 ± 14.8	FEV1(% predicted): 37.1 ± 12.9	NS	
Giardino (2004) (29)	I	20	FEV1(% predicted): 46 ± 16	FEV1(% predicted): 51 ± 17	NS	NA
Chau (2012) (59)	I	22	FEV1(% predicted): 33.59 ± 14.86	FEV1(% predicted): 33.64 ± 14.57	NS	Not reported
	C	18	FEV1(% predicted): 43.89 ± 29.11	FEV1(% predicted): 39.83 ± 15.36	NS	
Rutkowski (2021) (56)	I	25	FEV1%: 71.00 ± 23.66	FEV1%: 73.25 ± 23.24	NS	Not reported
	C	25	FEV1%: 86.48 ± 21.13	FEV1%: 90.24 ± 19.36	*p* < 0.049	
Clinical symptom						
Moy (2012) (25)	I	21	mMRC score: 2.48 ± 1.12	mMRC score: 3.22 ± 0.736	NS	NA
Tabak (2014) (47)	I	14	MRC score: 2.0 ± 0.9	MRC score: Changes after 3 weeks: −0.3 ± 0.7	NS	NS
	C	15	MRC score: 2.3 ± 1.4	MRC score: Changes after 3 weeks: −0.2 ± 0.9	NS	
Kawagoshi (2015) (31)	I	12	MRC score: 1.9 ± 0.8	MRC score: 1.2 ± 0.9	*p* = 0.039	Not reported
	C	15	MRC score: 1.9 ± 0.7	MRC score: 1.4 ± 0.9	NS	
Mendoza (2015) (37)	I	Baseline: 52 End: 50	CAT score: 15.5 ± 8.9	CAT score: Changes after 3 months: −3.5 ± 5.5	Not reported	*p* = 0.001
	C	Baseline: 50 End: 47	CAT score: 16.5 ± 7.3	CAT score: Changes after 3 months: −0.6 ± 6.6	Not reported	
Arbillaga-Etxarri (2018) (46)	I	132	CAT score: 12 ± 7	CAT score: 11 ± 7	*p* < 0.05	NS
	C	148	CAT score: 12 ± 8	CAT score: 11 ± 7	NS	
Jolly (2018) (38)	I	Baseline: 289 Month 6: 237 Month12: 247	MRC score: 1: 31% 2: 69%	MRC score: 1: 26% 2: 74%	Not reported	NS
	C	Baseline: 288 Month 6: 265 Month12: 269	MRC score: Month 6: 1 vs. 2 vs. 3 vs. 4 vs. 5: 32% vs. 58% vs. 6 3% vs. <1% Month 12: 1 vs. 2 vs. 3 vs. 4: 31% vs. 61% vs. 7 2%	MRC score: Month 6: 1 vs. 2 vs. 3 vs. 4: 32% vs. 60% vs. 7 2% Month 12: 1 vs. 2 vs. 3 vs. 4: 28% vs. 61% vs. 10 2%	Not reported	
O’Neill (2018) (28)	I	Baseline:23 End: 17	CAT score: 23.8 ± 6.9	CAT score: 22.5 ± 7.0	Not reported	Not reported
	C	Baseline:26 End: 19	CAT score: 18.7 ± 7.3	CAT score: 16.6 ± 5.3	Not reported	
Sutanto (2019) (53)	I	10	MRC score: 3(0.67)^*^	MRC score: 3(0.5)^*^	NS	*p* = 0.036
	C	10	MRC score: 2.5(0.5)^*^	MRC score: 2(0.4)^*^	NS	
Kohlbrenner (2020) (35)	I	Baseline: 37 End: 29	CAT score: 17.14 ± 6.77	CAT score: Changes after 3 months: −0.86 ± 5.18 Changes after 12 months: −1.32 ± 7.49	Not reported	NS
	C	Baseline: 37 End: 31	CAT score: 19.14 ± 6.15	CAT score: Changes after 3 months: −1.92 ± 4.45 Changes after 12 months: −1.63 ± 8.28	Not reported	
Park (2020) (33)	I	22	UCSD-SOBQ: 21.18 ± 16.05	UCSD-SOBQ: 21.45 ± 17.78	NS	NS
	C	20	UCSD-SOBQ: 19.25 ± 13.83	UCSD-SOBQ: 19.70 ± 14.34	NS	
Armstrong (2021) (27)	I	24	CAT score: 25.9 ± 6.4	CAT score: 21.7 ± 6.1	*p* = 0.001	*p* = 0.025
	C	24	CAT score: 27.0 ± 6.4	CAT score: 24.9 ± 7.1	*p* = 0.002	
Geidl (2021) (45)	I	167	CAT score: 23.23 ± 6.57	CAT score: Week 6: 17.82 ± 8.04 Month 6: 19.04 ± 7.99	Not reported	NS
	C	160	CAT score: 23.32 ± 6.82	CAT score: Week 6: 17.61 ± 7.74 Month 6: 18.93 ± 7.96	Not reported	
Robinson (2021) (26)	I	75	mMRC score: 2.0 ± 1.2	Changes after 3 months: −0.17 ± 1.82 Changes after 6 months: −0.06 ± 1,82	Changes after 3/6 months: Not reported	NS
	C	78	mMRC score: 2.1 ± 1.2	Changes after 3 months: 0.07 ± 1.85 Changes after 6 months: 0.00 ± 1.85	Changes after 3/6 months: Not reported	
Quality of life						
Giardino (2004) (29)	I	20	SGRQ: 50.1 ± 15.4	SGRQ: 41.9 ± 14.1	*p* < 0.01	NA
de Blok (2006) (41)	I	8	SGRQ: 59.1	SGRQ: 56.3	Not reported	NS
	C	8	SGRQ: 50.8	SGRQ: 44.7	Not reported	
Woo (2006) (48)	I	33	SGRQ: 53.69 ± 19.61	SGRQ: 34.72 ± 14.12	*p* < 0.001	NA
Wewel (2008) (23)	I	21	SGRQ: 59[51–64]^*^	SGRQ: 52[45–66]^*^	NS	NA
Hospes (2009) (32)	I	18	SGRQ: 37.7 ± 12.4	SGRQ: 34.2 ± 13.5	Not reported	*p* = 0.05
	C	17	SGRQ: 35.2 ± 18.7	SGRQ: 38.3 ± 16.8	Not reported	
Berry (2010) (14)	I	Baseline: 87 End: 61	CRQ: 4.3	CRQ: Month 3: 4.6 ± 0.1 Month 6: 4.5 ± 0.1 Month 12: 4.6 ± 0.1	Month 3, 12: *p* < 0.05	NS
	C	Baseline: 89 End: 69	CRQ: 4.3	CRQ: Month 3: 4.8 ± 0.1 Month 6: 4.7 ± 0.1 Month 12: 4.6 ± 0.1	Month 3, 12: *p* < 0.05	
Chau (2012) (59)	I	22	CRQ: Dyspnea: 4.27 ± 1.23 Fatigue: 4.09 ± 1.26 Emotion: 4.84 ± 1.47 Mastery: 4.60 ± 1.43	CRQ: Dyspnea: 3.97 ± 1.17 Fatigue: 4.11 ± 1.25 Emotion: 4.92 ± 1.40 Mastery: 4.61 ± 1.62	NS	NS
	C	18	CRQ: Dyspnea: 4.20 ± 0.83 Fatigue: 4.40 ± 0.99 Emotion: 5.24 ± 1.42 Mastery:4.94 ± 1.16	CRQ: Dyspnea: 4.45 ± 0.96 Fatigue: 4.79 ± 1.07 Emotion: 5.61 ± 1.17 Mastery:4.88 ± 1.27	NS	
Moy (2012) (25)	I	23	SF-36: 3.13 ± 0.757	SF-36: 3.22 ± 0.736	NS	NA
Song (2014) (64)	I	20	SGRQ: 52.9 ± 18.2	SGRQ: 42.3 ± 7.9	Not reported	*p* = 0.033
	C	20	SGRQ: 62.1 ± 17.4	SGRQ: 66.8 ± 6.4	Not reported	
Tabak (2014) (47)	I	14	CCQ: 2.0 ± 0.8	CCQ: Changes after 3 weeks: −0.3 ± 0.5	*p* = 0.046	NS
	C	15	CCQ: 1.8 ± 1.0	CCQ: Changes after 3 weeks: 0.0 ± 0.6	NS	
Altenburg (2015) (39)	I	Baseline: 78 Month 3: 65 Month15: 50	CRQ: 102[86–118]^*^	CRQ: Changes after 3 months: 4[−2–15]^*^ Changes after 15 months: 2[−6–10]^*^	Not reported	NS
	C	Baseline: 77 Month 3: 55 Month15: 51	CRQ: 109[87–119]^*^	CRQ: Changes after 3 months: 2[−7–14]^*^ Changes after 15 months: 2[−5–12]^*^	Not reported	
Kawagoshi (2015) (31)	I	12	CRQ: 98 ± 20	CRQ: 108 ± 19	*p* = 0.027	Not reported
	C	15	CRQ: 99 ± 19	CRQ: 110 ± 19	*p* < 0.01	
Mendoza (2015) (37)	I	Baseline: 52 End: 50	SGRQ: 41.9 ± 19.8	SGRQ: Changes after 3 months: −8.8 ± 12.2	Not reported	*p* = 0.02
	C	Baseline: 50 End: 47	SGRQ: 43.7 ± 16.7	SGRQ: Changes after 3 months: −3.8 ± 10.9	Not reported	
Cruz (2016) (44)	I	13	SGRQ: 31.5 ± 15.7	SGRQ: Month 3: 24.0 ± 13.6 Month 6: 23.1 ± 10.3	*p* < 0.001	NS
	C	13	SGRQ: 34.9 ± 14.7	SGRQ: Month 3: 26.9 ± 15.2 Month 6: 26.2 ± 15.3	*p* < 0.001	
Arbillaga-Etxarri (2018) (46)	I	132	CCQ: 1 ± 1	CCQ: 1 ± 1	*p* < 0.05	NS
	C	148	CCQ: 1 ± 1	CCQ: 1 ± 1	NS	
Jolly (2018) (38)	I	Baseline: 277 Month 6: 222 Month12: 217	SGRQ: 27.8 ± 14.6	SGRQ: Month 6: 28.6 ± 17.1 Month 12: 27.9 ± 15.7	Not reported	NS
	C	Baseline: 272 Month 6: 237 Month12: 256	SGRQ: 29.5 ± 14.5	SGRQ: Month 6: 30.5 ± 16.7 Month 12: 30.9 ± 17.0	Not reported	
O’Neill (2018) (28)	I	Baseline:23 End: 16	EQ-5D weighted health index: 0.5 ± 0.2 EQ-5D health state VAS: 56.2 ± 20.8	EQ-5D weighted health index: 0.5 ± 0.3 EQ-5D health state VAS: 58.6 ± 23.0	Not reported	Not reported
	C	Baseline:26 End: 19	EQ-5D weighted health index: 0.6 ± 0.3 EQ-5D health state VAS: 60.8 ± 12.3	EQ-5D weighted health index: 0.7 ± 0.2 EQ-5D health state VAS: 74.0 ± 19.9	Not reported	
Wootton (2018) (30)	I	49	SGRQ: 46 ± 18	SGRQ: Changes after 14 months: −5 Changes from 2 months to 14 months: 1	Changes after 14 months: *p* < 0.05	NS
	C	46	SGRQ: 47 ± 16	SGRQ: Changes after 14 months: −2 Changes from 2 months to 14 months: 4	Changes from 2 months to 14 months: *p* < 0.05	
Collins (2019) (40)	I	58	CRQ: Dyspnea: 16.6 ± 4.2 Fatigue: 15.9 ± 4.0 Emotion: 33.3 ± 7.9 Mastery: 18.8 ± 5.1	CRQ: Dyspnea: 21.1 ± 5.4 Fatigue: 17.7 ± 4.8 Emotion: 36.6 ± 8.6 Mastery: 21.2 ± 5.0	Not reported	Not reported^#^
	C	61	CRQ: Dyspnea: 16.2 ± 5.4 Fatigue: 16.5 ± 4.7 Emotion: 33.6 ± 9.4 Mastery: 19.6 ± 5.4	CRQ: Dyspnea: 19.4 ± 6.7 Fatigue: 18.4 ± 5.0 Emotion: 36.9 ± 9.0 Mastery: 21.0 ± 5.3	Not reported	
Sutanto (2019) (53)	I	10	SGRQ: 57.7 ± 11.6	SGRQ: 30.6 ± 5.9	*p* = 0.001	NS
	C	10	SGRQ: 54.1 ± 16.3	SGRQ: 29.4 ± 9.9	*p* = 0.002	
Jung (2020) (52)	I	10	CRQ: Dyspnea: 2.22 ± 1.09 Fatigue: 3.11 ± 1.43 Emotion: 3.85 ± 1.52 Mastery:3.83 ± 1.19	CRQ: Dyspnea: 2.96 ± 1.15 Fatigue: 3.27 ± 1.15 Emotion: 4.36 ± 1.01 Mastery:4.22 ± 0.74	Not reported	NA
Park (2020) (33)	I	22	SF-36: PCS: 43.43 ± 9.00 MCS: 51.62 ± 8.71	SF-36: PCS: 43.94 ± 8.97 MCS: 50.10 ± 8.33	NS	NS
	C	20	SF-36: PCS: 46.36 ± 5.58 MCS: 52.13 ± 8.49	SF-36: PCS: 44.95 ± 5.95 MCS: 49.03 ± 11.02	NS	
Yao (2020) (49)	I	64	Not reported	QLQ-C: Role function: 78.25 ± 2.41 Emotional function: 80.27 ± 2.38 Physical function: 80.43 ± 2.44 Cognitive function:79.91 ± 3.32 Social function: 80.15 ± 2.57	Not reported	*p* < 0.001
	C	64	Not reported	QLQ-C: Role function: 60.29 ± 2.11 Emotional function: 61.43 ± 2.39 Physical function: 61.02 ± 3.03 Cognitive function: 60.95 ± 2.86 Social function: 61.48 ± 2.43		
Armstrong (2021) (27)	I	24	CCQ: 2.5 ± 1.1	CCQ: 2.2 ± 1.1	NS	NS
	C	24	CCQ: 2.5 ± 1.3	CCQ: 2.4 ± 1.3	NS	
Criner (2021) (61)	I	Baseline: 67 End: 58	CCQ: 2.3 ± 0.91	Reported in diagram format	NS	Not reported
	C	Baseline: 70 End: 67	CCQ: 2.8 ± 0.97	Reported in diagram format	NS	
Geidl (2021) (45)	I	167	SGRQ: 55.5 ± 16.8	SGRQ: Week 6: 40.9 ± 21.6 Month 6: 44.3 ± 19.8	Not reported	NS
	C	160	SGRQ: 57.0 ± 17.1	SGRQ: Week 6: 40.4 ± 20.7 Month 6: 43.6 ± 20.5	Not reported	
Robinson (2021) (26)	I	75	SGRQ: 40.0 ± 15.3	Changes after 3 months: −14.63 ± 31.09 Changes after 6 months: −13.05 ± 31.09	Changes after 3/6 months: Not reported	NS
	C	78	SGRQ: 38.0 ± 17.8	Changes after 3 months: −13.86 ± 28.44 Changes after 6 months: −15.13 ± 28.44	Changes after 3/6 months: Not reported	
Norweg (2023) (36)	I	12	SGRQ: 52.47 ± 18.08	SGRQ: 40.72 ± 16.48	*p* = 0.01	Not reported
	C	3	SGRQ: 56.65 ± 11.79	SGRQ: 46.69 ± 0.85	Not reported	

I, intervention; C, control; NS, No significance; NA, Not applicable; 6WMD, 6-min walking distance; SGRQ, St. George Respiratory Questionnaire; QLQ-C30, Quality of Life Questionnaire-Core 30; ES-CA, Exercise of self-care agency; CRQ, Chronic Respiratory Questionnaire; MMAS-8, Morisky Medication Adherence Scale 8 items; CCQ, Clinical COPD Questionnaire; SF-36, Medical Outcome Study Short Form-36; PCS, Physical component subscale; MCS, Mental component subscale; ISWT, Incremental Shuttle Walk Test; MRC scale, Medical Research Council Dyspnea Scale; SEMCD, Self-Efficacy for Managing Chronic Disease 6-Item Scale; 1MSTS, 1-min sit-to-stand test; 2MST, 2-Minute Step Test; UCSD-SOBQ, The University of California, San Diego Shortness of Breath Questionnaire.

* Values are median values and quartiles (in parentheses).

# The total CRQ score was not reported.

**Table 5:** Effectiveness of nudge elements by delivery mode and targeted outcome.

	Medication adherence	Inhalation technique	Smoking cessation	Influenza vaccination	Physical activity	Exercise capacity	Self-efficacy	Pulmonary function	Clinical symptom	Quality of life
Social influence										
Mobile applications					1^a^	1^b^	2^b^			1^b^
Web-based										
Pedometer/devices										
Group sessions					1^a^	2^a^				2^c^
Text materials										
Gamification										
Mobile applications					1^a^					
Web-based						1^b^				
Pedometer/devices						4^c^		1^a^	1^b^	2^a^
Group sessions										
Text materials										
Reminder										
Mobile applications	2^b^									1^a^
Web-based										
Pedometer/devices								1^a^		1^a^
Group sessions										
Text materials	1^b^	1^b^		1^b^		1^a^	1^b^			1^b^
Feedback										
Mobile applications					1^a^				1^a^	1^a^
Web-based										
Pedometer/devices	3^b^		1^a^		17^c^	17^c^	6^c^	2^a^	11^c^	19^c^
Group sessions										
Text materials										

Numbers in the table represent the number of literatures corresponding to the intervention mode (vertical columns) and targeted outcome (horizontal columns).

a No statically significant outcome.

b Statically significant outcome.

c Mixed results.

In social influence interventions, exercise capacity, self-efficacy, and quality of life were significantly enhanced through mobile applications, while physical activity was ineffective. Using group sessions had inconsistent effects on quality of life and was ineffective for increasing exercise capacity or physical activity. Gamification intervention based on the web effectively increased exercise capacity. Physical activity was unaffected by gamification through mobile applications. Gamification using devices was beneficial for clinical symptoms but ineffective for lung function and quality of life, with conflicting results on exercise capacity.

Though they did not influence exercise capacity, reminders based on text materials were an excellent way to promote medication adherence, inhalation technique, influenza vaccination rates, self-efficacy, and quality of life. Mobile applications and devices that served as reminders had little effect on quality of life. Reminders via devices greatly increased medication adherence. However, mobile applications did not affect pulmonary function.

Through the chronolog, studies that employed feedback as a nudge to enhance medication adherence were effective. Pedometers and other devices had inconsistent impacts on quality of life, clinical symptoms, exercise capacity, self-efficacy, and physical activity, but had no effect on smoking cessation behavior or pulmonary function. Feedback via mobile applications did not influence the quality of life, clinical symptoms, or physical activity.

## 4 Discussion

To our best knowledge, this study is the first to summarize the evidence regarding the effectiveness of nudge in modifying patients with COPD and improving their health behaviors and outcomes. Prior research by Möllenkamp et al. (9) examined the effectiveness of nudges in enhancing patients’ ability to manage their chronic illnesses, including a subgroup of patients with COPD. However, this study only comprised two trials (14, 44) that were specifically focused on COPD and did not yield substantial conclusions. We included five different types of nudges for investigating the effects of various nudge types on the health behaviors and outcomes of patients with COPD, without restricting RCT studies. The health outcomes also exhibited considerable heterogeneity, including medication adherence, inhalation technique, smoking cessation, influenza vaccination, physical activity, self-efficacy, exercise capacity, pulmonary function, clinical symptoms, and quality of life. Owing to these variabilities, a qualitative synthesis that was consistent with the research by Möllenkamp et al. (9) and Kwan et al. (17) was carried out instead of a meta-analysis. Furthermore, many studies that used nudges might not have mentioned the term “nudge” explicitly because it was just introduced in 2008 (11). In order to expand the scope of inclusion and incorporate as much research on the nudging approach as feasible, our study included terms like social norm, social support, persuasion, and feedback in the search strategy.

Our systematic review found significant effects of nudging on medication adherence, inhalation technique, and vaccination behavior. Medication adherence was significantly improved by reminders via mobile applications or text materials, as well as feedback based on devices. Additionally, reminders through text materials greatly enhance inhalation techniques and vaccination behaviors in patients. The most recent *Global Initiative for Chronic Obstructive Lung Disease: 2023 Report* (1), emphasizes the importance that it is to promote medication adherence, inhalation techniques, vaccination, and other health-related behaviors to further enhance the health outcomes of patients with COPD. On the other hand, low-cost, simple nudging interventions—like using a simple mobile application, equipped with devices such as the chronolog, offering text materials or cards, or simply labeling on inhalation devices—can significantly improve health behaviors and benefit patients with COPD.

Comparisons of intervention types revealed that feedback was the most frequently investigated nudge, followed by gamification, reminders, and social influence. According to our review, nudge-based interventions were found to increase medication adherence, inhalation technique, and vaccine behavior, but not lung function or smoking cessation behavior. It is evident from other outcomes that the type of nudging and the delivery modes have an impact on the results. For instance, social influence had a strong impact, but reminders and feedback were ineffective when mobile applications were utilized as delivery modes to affect the quality of life (47, 49, 61). Reminders sent via text materials significantly improved quality of life (64), whereas no discernible effects were observed with mobile applications or devices (59, 61), suggesting that delivery modes might have a significant impact on the effectiveness given the parallels in study design and patient variables. We were unable to make reliable conclusions regarding the relationship between modes of delivery and health outcomes due to the small number of studies. Hence, more research is required to confirm the impact of different types of nudging on the health behavior outcomes of patients with COPD.

Conflicting outcomes in studies investigating interventions for COPD may be attributable to variations in patient characteristics, such as baseline disease severity and levels of physical activity. For example, Mendoza et al. (37) reported that 80% of participants in their study had mild to moderate COPD, and the use of pedometers was associated with improvements in quality of life, clinical symptoms, exercise capacity, and physical activity. In contrast, a similar intervention yielded no significant effects on physical activity, exercise endurance, or clinical symptoms among patients with severe to very severe COPD (35). Furthermore, Geidl et al. (45) found that patients with high baseline levels of physical activity did not benefit from the intervention in terms of increasing physical activity or improving clinical symptoms. Armstrong et al. (27), however, observed notable improvements in physical activity and clinical symptoms in patients using pedometers as part of their intervention. Interestingly, in the study by Moy et al. (25), more than half of the patients exhibited good baseline health and minimal dyspnea, and the intervention failed to produce significant changes in clinical symptoms or quality of life.

In addition, the duration and season of the interventions may also affect intervention effectiveness. Due to the short intervention period of some studies, statistical significance was not observed for some outcome measures that could only measure significant changes after long-term intervention, such as lung function (29, 42, 56, 59). Since the study by de Blok et al. (41) was carried out in autumn and wintertime, the physical activity of patients did not improve during this time, which made it even more challenging to assess if the nudge was beneficial. Future research should take into account the duration time of the intervention and seasons that would affect the health outcomes of patients with COPD.

The extent of the change in patients’ health behaviors depends on the study design. Several studies have reported the same effects of nudges on patients’ health outcomes, but the conclusions were different. For example, pedometers were utilized in all of these studies as feedback tools to encourage physical activity of patients. The study from Wewel et al. (23) that lacked a control group showed that the physical activity and exercise capacity significantly improved in the intervention group. Similar intervention regimens were employed in other research (45, 46), but no substantial improvements in the outcomes were noted. In the absence of a control group, there is no reason to rule out the possibility that routine care is responsible for improvements in health outcomes. For instance, the study by Geidl et al. (45) indicated that while patients in the control group merely got pulmonary rehabilitation without any nudging intervention, they still exhibited significant changes over time in parameters such as physical activity. A similar result was also observed in the comparison between Berry et al. (14) and Woo et al. (48).

### 4.1 Limitations

There are several limitations to this study. First, the classification of interventions that fall within the notion of nudge has not yet been widely accepted (11). Numerous studies that supported the nudging theory failed to use this phrase explicitly. To broaden the scope of our search, we chose potentially pertinent research, which also made our search formula not concise. Second, despite having sufficient RCTs (31 of 43), due to the high heterogeneity of the type of intervention, included outcome variables, and study designs, quantitative meta-analysis to examine the intervention effects of nudges is not feasible, so we had to choose the vote counting method as the last thing for narrative synthesis, which did not allow us to critically quantify the effect of nudges. Third, the validity of the results of some studies may be impacted by the small sample size or inadequate statistical power. Last, 15 out of the 43 publications were found to have a high risk of bias. These findings were consistent with those made by Kwan et al. (17) and were related to selection biases, differences in baseline characteristics and outcomes, high attrition rates, and lack of blinded assessment. Although it is currently unknown how these biases affect the way interventions based on nudge are carried out, we did not rule out this research due to its significance in real-world circumstances.

### 4.2 Implications for future research

This study provides a novel viewpoint on COPD management from the standpoint of behavioral economics since it is the first to explore the application of nudge theory and strategies to the health behaviors and outcomes of patients with COPD. Though the theory is still in its infancy and research on the efficacy of interventions is also still in its early phases, we believe the theory has a promising future. To further examine the impact of nudge in the management of chronic diseases such as COPD, future studies may need to take into account the following points.

Firstly, assuming that academics have come to a consensus on the classification of nudges, we may attempt, incrementally, to examine the individual intervention effect of a particular type of nudge or narrow our emphasis to a single health behavior or result. Whenever feasible, using randomized controlled trials (RCTs) for study design and quantitative meta-analysis may reach more reliable conclusions.

Secondly, to assess the effects of nudges, aim to conduct research in the same context to test their effects, including selecting subjects with the same characteristics, such as age, gender, ethnicity, and even COPD stage. Also consider the impact of the delivery mode of the nudge, the duration and frequency of the intervention, and the season on the effectiveness of the intervention. Performing subgroup analyses based on patient characteristics or intervention delivery mode, etc., might also support the study.

Lastly, a more thorough exploration of the degree to which nudge interventions are effective in their own right requires the exclusion of the confounding effects of other concurrent non-nudge interventions such as education, training, general encouragement, and traditional pulmonary rehabilitation.

## 5 Conclusion

We have delineated a list of nudging interventions for patients with COPD, including social influence, gamification, reminders, and feedback, which provides a new approach and strategy for COPD management. In particular, medication adherence was significantly improved by reminders via mobile applications (60, 61) or text materials (64), as well as feedback based on devices. Reminders through text materials also greatly enhance inhalation techniques and vaccination behaviors in patients, which can be extensively advocated and employed in healthcare settings. Furthermore, we propose factors such as the delivery modes, the baseline characteristics of patients, and the duration and seasons of interventions that could affect the effectiveness of interventions. By employing the same background settings, further research might verify the impacts of various nudging strategies. It is also important to design the type of study as appropriately as possible.

## Data Availability

The original contributions presented in the study are included in the article/Supplementary material, further inquiries can be directed to the corresponding authors.
