# Supplementary material for: Nudge theories and strategies influencing adult health behaviors and outcomes in COPD management: a systematic review
Source: Front Public Health. 2024 Nov 5;12:1404590. doi: 10.3389/fpubh.2024.1404590 (PMC11573774; doi:10.3389/fpubh.2024.1404590)
Supplement: Supplementary file 1 [file Table_1.DOCX]

Supplementary Material

# Supplementary Table 1 Search syntax

**Database: PubMed**

**Rundate: 26/06/2023**

((((((((("Pulmonary Disease, Chronic Obstructive"[Mesh]) OR ("Chronic Obstructive Lung Disease"[Title/Abstract])) OR ("Chronic Obstructive Pulmonary Disease*"[Title/Abstract])) OR ("Chronic Obstructive Airway Disease"[Title/Abstract])) OR ("Airflow Obstruction*, Chronic"[Title/Abstract])) OR ("Chronic Airflow Obstruction*"[Title/Abstract])) OR (COAD[Title/Abstract])) OR (COPD[Title/Abstract]))

AND

(((((((((((((((((((((((((((((((((("Social Norms"[Mesh] OR "Health Communication"[Mesh] OR "Social Support"[Mesh] OR "Persuasive Communication"[Mesh] OR "Feedback"[Mesh] OR "Cues"[Mesh] OR "Peer Influence"[Mesh] OR "Heuristics"[Mesh]) OR (Nudg*[Title/Abstract])) OR (E-nudg*[Title/Abstract])) OR ("Choice architect*"[Title/Abstract])) OR (Encourag*[Title/Abstract])) OR (Motivat*[Title/Abstract])) OR (Persua*[Title/Abstract])) OR ("Risk communication"[Title/Abstract])) OR ("Health Communications"[Title/Abstract])) OR ("Communication*, Health"[Title/Abstract])) OR ("Communication, Persuasive"[Title/Abstract])) OR (Brainwashing[Title/Abstract])) OR (Cue[Title/Abstract])) OR (Heuristic[Title/Abstract])) OR ("Rule of Thumb"[Title/Abstract])) OR (Incentive*[Title/Abstract])) OR (Remind*[Title/Abstract])) OR ("Norm*, Social"[Title/Abstract])) OR ("Social Norm"[Title/Abstract])) OR ("Norm*, Societal"[Title/Abstract])) OR ("Societal Norm*"[Title/Abstract])) OR ("Support, Social"[Title/Abstract])) OR ("Social Care"[Title/Abstract])) OR ("Care, Social"[Title/Abstract])) OR ("Online Social Support*"[Title/Abstract])) OR ("Social Support*, Online"[Title/Abstract])) OR ("Support, Online Social"[Title/Abstract])) OR ("Perceived Social Support*"[Title/Abstract])) OR ("Social Support*, Perceived"[Title/Abstract])) OR ("Support*, Perceived Social"[Title/Abstract])) OR ("Influence, Peer"[Title/Abstract])) OR ("Peer Pressure"[Title/Abstract])) OR ("Pressure, Peer"[Title/Abstract])) OR (Feedbacks[Title/Abstract])))

AND

((((((((((((((("Treatment Adherence and Compliance"[Mesh]) OR "Disease Management"[Mesh]) OR ( "Medication Adherence"[Mesh] OR "Behavior Control"[Mesh] OR "Health Behavior"[Mesh] OR "Patient Compliance"[Mesh] OR "Choice Behavior"[Mesh] OR "Attitude to Health"[Mesh] OR "Self-Management"[Mesh] )) OR (Behavior*[Title/Abstract])) OR (Behaviour*[Title/Abstract])) OR (Self-care[Title/Abstract])) OR ("Self management"[Title/Abstract])) OR ("Management, Self"[Title/Abstract])) OR ("Disease Managements"[Title/Abstract])) OR ("Management*, Disease"[Title/Abstract])) OR ("Health, Attitude to"[Title/Abstract])) OR ("Health Attitude*"[Title/Abstract])) OR ("Attitude*, Health"[Title/Abstract])) OR ("Lifestyle modification"[Title/Abstract])) OR (Adhere*[Title/Abstract]))

Filters: English

**Results: 781**

**Database: Embase**

**Rundate: 26/06/2023**

| #1 | 'chronic obstructive lung disease':ab,ti,kw OR 'chronic obstructive pulmonary disease*':ab,ti,kw OR coad:ab,ti,kw OR copd:ab,ti,kw OR 'chronic obstructive airway disease':ab,ti,kw OR 'airflow obstruction*, chronic':ab,ti,kw OR 'chronic airflow obstruction*':ab,ti,kw OR 'pulmonary disease, chronic obstructive'/exp OR 'pulmonary disease, chronic obstructive' |
| --- | --- |
| #2 | 'persuasive communication'/exp OR 'health communication'/exp OR 'cues'/exp OR 'heuristics'/exp OR 'social norms'/exp OR 'social support'/exp OR 'peer influence'/exp OR 'feedback'/exp OR nudg*:ab,ti,kw OR 'e nudg*':ab,ti,kw OR 'choice architect*':ab,ti,kw OR encourag*:ab,ti,kw OR motivat*:ab,ti,kw OR persua*:ab,ti,kw OR 'risk communication':ab,ti,kw OR 'health communications':ab,ti,kw OR 'communication*, health':ab,ti,kw OR 'communication, persuasive':ab,ti,kw OR brainwashing:ab,ti,kw OR cue:ab,ti,kw OR heuristic:ab,ti,kw OR 'rule of thumb':ab,ti,kw OR incentive*:ab,ti,kw OR remind*:ab,ti,kw OR 'norm*, social':ab,ti,kw OR 'social norm':ab,ti,kw OR 'norm*, societal':ab,ti,kw OR 'societal norm*':ab,ti,kw OR 'support, social':ab,ti,kw OR 'social care':ab,ti,kw OR 'care, social':ab,ti,kw OR 'online social support*':ab,ti,kw OR 'social support*, online':ab,ti,kw OR 'support, online social':ab,ti,kw OR 'perceived social support*':ab,ti,kw OR 'social support*, perceived':ab,ti,kw OR 'support*, perceived social':ab,ti,kw OR 'influence, peer':ab,ti,kw OR 'peer pressure':ab,ti,kw OR 'pressure, peer':ab,ti,kw OR 'feedbacks':ab,ti,kw |
| #3 | 'choice behavior'/exp OR 'self-management'/exp OR 'disease management'/exp OR 'attitude to health'/exp OR 'health behavior'/exp OR 'patient compliance'/exp OR 'treatment adherence and compliance'/exp OR 'medication adherence'/exp OR 'behavior control'/exp OR behavior*:ab,ti,kw OR behaviour*:ab,ti,kw OR 'self care':ab,ti,kw OR 'self management':ab,ti,kw OR 'management, self':ab,ti,kw OR 'disease managements':ab,ti,kw OR 'management*, disease':ab,ti,kw OR 'health, attitude to':ab,ti,kw OR 'health attitude*':ab,ti,kw OR 'attitude*, health':ab,ti,kw OR 'lifestyle modification':ab,ti,kw OR adhere*:ab,ti,kw |
| #4 | #1 AND #2 AND #3 AND [english]/lim |

**Results: 2975**

**Database: Cochrane**

**Rundate: 26/06/2023**

| #1 | ("Chronic Obstructive Lung Disease"):ti,ab,kw OR ("Chronic Obstructive Pulmonary Disease"):ti,ab,kw OR ("Chronic Obstructive Airway Disease"):ti,ab,kw OR ("Airflow Obstruction, Chronic"):ti,ab,kw OR ("Chronic Airflow Obstruction"):ti,ab,kw OR ("Chronic Obstructive Pulmonary Diseases"):ti,ab,kw OR ("Airflow Obstructions, Chronic"):ti,ab,kw OR ("Chronic Airflow Obstructions"):ti,ab,kw OR (COAD):ti,ab,kw OR (COPD):ti,ab,kw |
| --- | --- |
| #2 | MeSH descriptor: [Pulmonary Disease, Chronic Obstructive] explode all trees |
| #3 | #1 OR #2 |
| #4 | MeSH descriptor: [Cues] explode all trees |
| #5 | MeSH descriptor: [Persuasive Communication] explode all trees |
| #6 | MeSH descriptor: [Health Communication] explode all trees |
| #7 | MeSH descriptor: [Heuristics] explode all trees |
| #8 | MeSH descriptor: [Social Norms] explode all trees |
| #9 | MeSH descriptor: [Social Support] explode all trees |
| #10 | MeSH descriptor: [Peer Influence] explode all trees |
| #11 | MeSH descriptor: [Feedback] explode all trees |
| #12 | (Nudg*):ti,ab,kw OR (E-nudg*):ti,ab,kw OR ("Choice architecture"):ti,ab,kw OR (Encourag*):ti,ab,kw OR (Motivat*):ti,ab,kw OR (Persua*):ti,ab,kw OR ("Risk communication"):ti,ab,kw OR ("Health Communications"):ti,ab,kw OR ("Communication, Health"):ti,ab,kw OR ("Communication, Persuasive"):ti,ab,kw OR ("Communications, Health"):ti,ab,kw OR (Brainwashing):ti,ab,kw OR (Cue):ti,ab,kw OR (Heuristic):ti,ab,kw OR ("Rule of Thumb"):ti,ab,kw OR (Incentive*):ti,ab,kw OR (Remind*):ti,ab,kw OR ("Norm, Social"):ti,ab,kw OR ("Norms, Social"):ti,ab,kw OR ("Social Norm"):ti,ab,kw OR ("Norm, Societal"):ti,ab,kw OR ("Norms, Societal"):ti,ab,kw OR ("Societal Norm"):ti,ab,kw OR ("Societal Norms"):ti,ab,kw OR ("Support, Social"):ti,ab,kw OR ("Social Care"):ti,ab,kw OR ("Care, Social"):ti,ab,kw OR ("Online Social Support"):ti,ab,kw OR ("Online Social Supports"):ti,ab,kw OR ("Social Support, Online"):ti,ab,kw OR ("Social Supports, Online"):ti,ab,kw OR ("Support, Online Social"):ti,ab,kw OR ("Perceived Social Support"):ti,ab,kw OR ("Perceived Social Supports"):ti,ab,kw OR ("Social Support, Perceived"):ti,ab,kw OR ("Social Supports, Perceived"):ti,ab,kw OR ("Support, Perceived Social"):ti,ab,kw OR ("Supports, Perceived Social"):ti,ab,kw OR ("Influence, Peer"):ti,ab,kw OR ("Peer Pressure"):ti,ab,kw OR ("Pressure, Peer"):ti,ab,kw OR (Feedbacks):ti,ab,kw |
| #13 | #4 OR #5 OR #6 OR #7 OR #8 OR #9 OR #10 OR #11 OR #12 |
| #14 | MeSH descriptor: [Choice Behavior] explode all trees |
| #15 | MeSH descriptor: [Self-Management] explode all trees |
| #16 | MeSH descriptor: [Disease Management] explode all trees |
| #17 | MeSH descriptor: [Attitude to Health] explode all trees |
| #18 | MeSH descriptor: [Health Behavior] explode all trees |
| #19 | MeSH descriptor: [Patient Compliance] explode all trees |
| #20 | MeSH descriptor: [Treatment Adherence and Compliance] explode all trees |
| #21 | MeSH descriptor: [Medication Adherence] explode all trees |
| #22 | MeSH descriptor: [Behavior Control] explode all trees |
| #23 | (Behavior*):ti,ab,kw OR (Behaviour*):ti,ab,kw OR (Self-care):ti,ab,kw OR ("Self management"):ti,ab,kw OR ("Management, Self"):ti,ab,kw OR ("Disease Managements"):ti,ab,kw OR ("Management, Disease"):ti,ab,kw OR ("Managements, Disease"):ti,ab,kw OR ("Health, Attitude to"):ti,ab,kw OR ("Health Attitude"):ti,ab,kw OR ("Health Attitudes"):ti,ab,kw OR ("Attitude, Health"):ti,ab,kw OR ("Attitudes, Health"):ti,ab,kw OR ("Lifestyle modification"):ti,ab,kw OR (Adhere*):ti,ab,kw |
| #24 | #14 OR #15 OR #16 OR #17 OR #18 OR #19 OR #20 OR #21 OR #22 OR #23 |
| #25 | #3 AND #13 AND #24 |
| #26 | English:la |
| #27 | #25 AND #26 |

**Results (Trials): 417**

**Database: Scopus**

**Rundate: 26/06/2023**

( ( TITLE-ABS-KEY ( "Pulmonary Disease, Chronic Obstructive" ) OR TITLE-ABS-KEY ( "Chronic Obstructive Lung Disease" ) OR TITLE-ABS-KEY ( "Chronic Obstructive Pulmonary Disease*" ) OR TITLE-ABS-KEY ( "Chronic Obstructive Airway Disease" ) OR TITLE-ABS-KEY ( "Airflow Obstruction*, Chronic" ) OR TITLE-ABS-KEY ( "Chronic Airflow Obstruction*" ) OR TITLE-ABS-KEY ( coad ) OR TITLE-ABS-KEY ( copd ) ) )

AND

( ( ( TITLE-ABS-KEY ( nudg* ) OR TITLE-ABS-KEY ( e-nudg* ) OR TITLE-ABS-KEY ( "Choice architect*" ) OR TITLE-ABS-KEY ( encourag* ) OR TITLE-ABS-KEY ( motivat* ) OR TITLE-ABS-KEY ( persua* ) OR TITLE-ABS-KEY ( "Persuasive communication" ) OR TITLE-ABS-KEY ( "Communication, Persuasive" ) OR TITLE-ABS-KEY ( "Risk communication" ) OR TITLE-ABS-KEY ( "Health communication*" ) OR TITLE-ABS-KEY ( "Communication*, Health" ) OR TITLE-ABS-KEY ( "Rule of Thumb" ) OR TITLE-ABS-KEY ( brainwashing ) ) ) OR ( ( TITLE-ABS-KEY ( cue* ) OR TITLE-ABS-KEY ( heuristic* ) OR TITLE-ABS-KEY ( "Social norms" ) OR TITLE-ABS-KEY ( "Social support" ) OR TITLE-ABS-KEY ( "Peer influence" ) OR TITLE-ABS-KEY ( feedback* ) OR TITLE-ABS-KEY ( incentive* ) OR TITLE-ABS-KEY ( remind* ) OR TITLE-ABS-KEY ( "Norm*, Social" ) OR TITLE-ABS-KEY ( "Social Norm" ) OR TITLE-ABS-KEY ( "Norm*, Societal" ) OR TITLE-ABS-KEY ( "Societal Norm*" ) OR TITLE-ABS-KEY ( "Support, Social" ) OR TITLE-ABS-KEY ( "Social Care" ) OR TITLE-ABS-KEY ( "Care, Social" ) OR TITLE-ABS-KEY ( "Online Social Support*" ) OR TITLE-ABS-KEY ( "Social Support*, Online" ) OR TITLE-ABS-KEY ( "Support, Online Social" ) OR TITLE-ABS-KEY ( "Perceived Social Support*" ) OR TITLE-ABS-KEY ( "Social Support*, Perceived" ) OR TITLE-ABS-KEY ( "Support*, Perceived Social" ) OR TITLE-ABS-KEY ( "Influence, Peer" ) OR TITLE-ABS-KEY ( "Peer Pressure" ) OR TITLE-ABS-KEY ( "Pressure, Peer" ) ) ) )

AND

( ( TITLE-ABS-KEY ( "Choice behavior" ) OR TITLE-ABS-KEY ( self-care ) OR TITLE-ABS-KEY ( self-management ) OR TITLE-ABS-KEY ( "Self management" ) OR TITLE-ABS-KEY ( "Management, Self" ) OR TITLE-ABS-KEY ( "Disease management" ) OR TITLE-ABS-KEY ( "Disease Managements" ) OR TITLE-ABS-KEY ( "Management*, Disease" ) OR TITLE-ABS-KEY ( "Attitude to health" ) OR TITLE-ABS-KEY ( "Health, Attitude to" ) OR TITLE-ABS-KEY ( "Health Attitude*" ) OR TITLE-ABS-KEY ( "Attitude*, Health" ) OR TITLE-ABS-KEY ( "Health behavior" ) OR TITLE-ABS-KEY ( "Patient compliance" ) OR TITLE-ABS-KEY ( "Treatment Adherence and Compliance" ) OR TITLE-ABS-KEY ( "Medication Adherence" ) OR TITLE-ABS-KEY ( "Behavior control" ) OR TITLE-ABS-KEY ( behavior* ) OR TITLE-ABS-KEY ( behaviour* ) OR TITLE-ABS-KEY ( "Lifestyle modification" ) OR TITLE-ABS-KEY ( adhere* ) ) )

AND

( LIMIT-TO ( LANGUAGE , "English" ) )

**Results: 1602**

**Database: Web of Science**

**Rundate: 26/06/2023**

| #1 | (((((((TS=("Pulmonary Disease, Chronic Obstructive")) OR TS=("Chronic Obstructive Lung Disease")) OR TS=("Chronic Obstructive Pulmonary Disease*")) OR TS=("Chronic Obstructive Airway Disease")) OR TS=("Airflow Obstruction*, Chronic")) OR TS=("Chronic Airflow Obstruction*")) OR TS=(COAD)) OR TS=(COPD) |
| --- | --- |
| #2 | ((((((((((((((((((((((((((((((((((((TS=(Nudg*)) OR TS=(E-nudg*)) OR TS=("Choice architect*")) OR TS=(Encourag*)) OR TS=(Motivat*)) OR TS=(Persua*)) OR TS=("Persuasive communication")) OR TS=("Communication, Persuasive")) OR TS=("Risk communication")) OR TS=("Health communication*")) OR TS=("Communication*, Health")) OR TS=("Rule of Thumb")) OR TS=(Brainwashing)) OR TS=(Cue*)) OR TS=(Heuristic*)) OR TS=("Social norms")) OR TS=("Social support")) OR TS=("Peer influence")) OR TS=(Feedback*)) OR TS=(Incentive*)) OR TS=(Remind*)) OR TS=("Norm*, Social")) OR TS=("Social Norm")) OR TS=("Norm*, Societal")) OR TS=("Societal Norm*")) OR TS=("Support, Social")) OR TS=("Social Care")) OR TS=("Care, Social")) OR TS=("Online Social Support*")) OR TS=("Social Support*, Online")) OR TS=("Support, Online Social")) OR TS=("Perceived Social Support*")) OR TS=("Social Support*, Perceived")) OR TS=("Support*, Perceived Social")) OR TS=("Influence, Peer")) OR TS=("Peer Pressure")) OR TS=("Pressure, Peer") |
| #3 | ((((((((((((((((((((TS=("Choice behavior")) OR TS=(Self-care)) OR TS=(Self-management)) OR TS=("Self management")) OR TS=("Management, Self")) OR TS=("Disease management")) OR TS=("Disease Managements")) OR TS=("Management*, Disease")) OR TS=("Attitude to health")) OR TS=("Health, Attitude to")) OR TS=("Health Attitude*")) OR TS=("Attitude*, Health")) OR TS=("Health behavior")) OR TS=("Patient compliance")) OR TS=("Treatment Adherence and Compliance")) OR TS=("Medication Adherence")) OR TS=("Behavior control")) OR TS=(Behavior*)) OR TS=(Behaviour*)) OR TS=("Lifestyle modification")) OR TS=(Adhere*) |
| #4 | #1 AND #2 AND #3 AND and English(Language) |

**Results: 1247**

**Database: PsycInfo (ESBCO)**

**Rundate: 26/06/2023**

| S1 | AB "Pulmonary Disease, Chronic Obstructive" OR AB "Chronic Obstructive Lung Disease" OR AB "Chronic Obstructive Pulmonary Disease*" OR AB "Chronic Obstructive Airway Disease" OR AB "Airflow Obstruction*, Chronic" OR AB "Chronic Airflow Obstruction*"OR AB COAD OR AB COPD |
| --- | --- |
| S2 | AB Nudg* OR AB E-nudg* OR AB "Choice architect*" OR AB Encourag* OR AB Motivat* OR AB Persua* OR AB "Persuasive communication" OR AB "Communication, Persuasive" OR AB "Risk communication" OR AB "Health communication*" ORAB "Communication*, Health" OR AB "Rule of Thumb" AB Brainwashing OR AB Cue* OR AB Heuristic* OR AB "Social norms" OR AB "Social support" OR AB "Peer influence" OR AB Feedback* OR AB Incentive* OR AB Remind* OR AB "Norm*, Social" OR AB "Social Norm" OR AB "Norm*, Societal" AB "Societal Norm*" OR AB "Support, Social" OR AB "Social Care" OR AB "Care, Social" OR AB "Online Social Support*"OR AB "Social Support*, Online" ORAB "Support, Online Social" OR AB "Perceived Social Support*"OR AB "Social Support*, Perceived" OR AB "Support*, Perceived Social" OR AB "Influence, Peer" OR AB "Peer Pressure" OR AB " Pressure, Peer" |
| S3 | AB "Health behavior" OR AB "Patient compliance" OR AB ( "Treatment Adherence and Compliance" ) OR AB "Medication Adherence" OR AB "Behavior control" OR AB Behavior* OR AB Behaviour* OR AB "Lifestyle modification" OR AB Adhere* OR AB "Choice behavior" OR AB Self-care OR AB Self-management OR AB "Self management" OR AB "Management, Self" OR AB "Disease management" OR AB "Disease Managements" OR AB "Management*, Disease" OR AB "Attitude to health" OR AB "Health, Attitude to" OR AB "Health Attitude*" OR AB "Attitude*, Health" |
| S4 | S1 AND S2 AND S3 |

Limits: English

**Results: 118**

**Database: EconLit (ESBCO)**

**Rundate: 26/06/2023**

| S1 | AB "Pulmonary Disease, Chronic Obstructive" OR AB "Chronic Obstructive Lung Disease" OR AB "Chronic Obstructive Pulmonary Disease*" OR AB "Chronic Obstructive Airway Disease" OR AB "Airflow Obstruction*, Chronic" OR AB "Chronic Airflow Obstruction*"OR AB COAD OR AB COPD |
| --- | --- |
| S2 | AB Nudg* OR AB E-nudg* OR AB "Choice architect*" OR AB Encourag* OR AB Motivat* OR AB Persua* OR AB "Persuasive communication" OR AB "Communication, Persuasive" OR AB "Risk communication" OR AB "Health communication*" ORAB "Communication*, Health" OR AB "Rule of Thumb" AB Brainwashing OR AB Cue* OR AB Heuristic* OR AB "Social norms" OR AB "Social support" OR AB "Peer influence" OR AB Feedback* OR AB Incentive* OR AB Remind* OR AB "Norm*, Social" OR AB "Social Norm" OR AB "Norm*, Societal" AB "Societal Norm*" OR AB "Support, Social" OR AB "Social Care" OR AB "Care, Social" OR AB "Online Social Support*"OR AB "Social Support*, Online" ORAB "Support, Online Social" OR AB "Perceived Social Support*"OR AB "Social Support*, Perceived" OR AB "Support*, Perceived Social" OR AB "Influence, Peer" OR AB "Peer Pressure" OR AB " Pressure, Peer" |
| S3 | AB "Health behavior" OR AB "Patient compliance" OR AB ( "Treatment Adherence and Compliance" ) OR AB "Medication Adherence" OR AB "Behavior control" OR AB Behavior* OR AB Behaviour* OR AB "Lifestyle modification" OR AB Adhere* OR AB "Choice behavior" OR AB Self-care OR AB Self-management OR AB "Self management" OR AB "Management, Self" OR AB "Disease management" OR AB "Disease Managements" OR AB "Management*, Disease" OR AB "Attitude to health" OR AB "Health, Attitude to" OR AB "Health Attitude*" OR AB "Attitude*, Health" |
| S4 | S1 AND S2 AND S3 |

Limits: English

**Results: 1**

# Supplementary Table 2 Characteristics of study participants

| First Author  (year) | Title | Country | N | Mean age (years) | Male  (%) |
| --- | --- | --- | --- | --- | --- |
| Altenburg (2015) | Short- and long-term effects of a physical activity counselling programme in COPD: a randomized controlled trial | Netherlands | 155 | 62 | 66 |
| Arbillaga-Etxarri (2018) | Long-term efficacy and effectiveness of a behavioural and community-based exercise intervention (Urban Training) to increase physical activity in patients with COPD: a randomised controlled trial | Spain | 407 | 69±8 | 87 |
| Armstrong (2021) | Behavioural modification interventions alongside pulmonary rehabilitation improve COPD patients' experiences of physical activity | UK | 60 | 72±9 | 37.5 |
| Berry (2010) | A Lifestyle Activity Intervention in Patients with Chronic Obstructive Pulmonary Disease | USA | 176 | 66±10 | 54 |
| Burkow (2018) | Promoting exercise training and physical activity in daily life: a feasibility study of a virtual group intervention for behaviour change in COPD | Norway | 10 | 65.7 | 30 |
| Chau (2012) | A feasibility study to investigate the acceptability and potential effectiveness of a telecare service for older people with chronic obstructive pulmonary disease | China | 53 | 72.93±6.04 | 97.5 |
| Collins (2019) | The Effect of Breathing Retraining Using Metronome-Based Acoustic Feedback on Exercise Endurance in COPD: A Randomized Trial | USA | 119 | 66±8 | 95 |
| Colombo (2023) | A virtual reality-based endurance training program for COPD patients: acceptability and user experience | Italy | 14 | 70.92±7.84 | 50 |
| Criner (2021) | The Impact of Budesonide/Formoterol pMDI Medication Reminders on Adherence in Chronic Obstructive Pulmonary Disease (COPD) Patients: Results of a Randomized, Phase 4, Clinical Study | USA | 137 | 66.7±8.44 | 56 |
| Cruz (2014) | Impact of feedback on physical activity levels of individuals with chronic obstructive pulmonary disease during pulmonary rehabilitation: A feasibility study | Portugal | 16 | 65.63±10.57 | 68.8 |
| Cruz (2016) | Walk2Bactive: A randomised controlled trial of a physical activity-focused behavioural intervention beyond pulmonary rehabilitation in chronic obstructive pulmonary disease | Portugal | 32 | 66.4±8.4 | 84 |
| de Blok (2006) | The effects of a lifestyle physical activity counseling program with feedback of a pedometer during pulmonary rehabilitation in patients with COPD: A pilot study | Netherlands | 21 | 64.0±11.3 | 43 |
| Esteve (1996) | The effects of breathing pattern training on ventilatory function in patients with COPD | France | 20 | 71.8±11.8 | Not reported |
| Geidl (2021) | Long-term benefits of adding a pedometer to pulmonary rehabilitation for copd: The randomized controlled star trial | Germany | 327 | 58.02±5.43 | 69 |
| Giardino (2004) | Combined heart rate variability and pulse oximetry biofeedback for chronic obstructive pulmonary disease: Preliminary findings | USA | 20 | 63±9.6 | 50 |
| Hospes (2009) | Enhancement of daily physical activity increases physical fitness of outclinic COPD patients: Results of an exercise counseling program | Netherlands | 39 | 62 | 60 |
| Jolly (2018) | Self management of patients with mild COPD in primary care: Randomised controlled trial | UK | 577 | 70.4 | 63 |
| Jung (2020) | A Virtual Reality-Supported Intervention for Pulmonary Rehabilitation of Patients With Chronic Obstructive Pulmonary Disease: Mixed Methods Study | UK | 10 | 68.9 | 60 |
| Kawagoshi (2015) | Effects of low-intensity exercise and home-based pulmonary rehabilitation with pedometer feedback on physical activity in elderly patients with chronic obstructive pulmonary disease | Japan | 39 | 74±8 | 89 |
| Kohlbrenner (2020) | Long-term effects of pedometer-based physical activity coaching in severe copd: A randomized controlled trial | Switzerland | 74 | 66±9 | 68 |
| Mendoza (2015) | Pedometers to enhance physical activity in COPD: A randomised controlled trial | Chile | 102 | 68.7±8.5 | 60.8 |
| Morfaw (2023) | Improvement of non-adherence to medication in adult COPD patients using medisafe medication management mobile | USA | 30 | 52.6 | 67 |
| Moy (2012) | A pilot study of an Internet walking program and pedometer in COPD | USA | 27 | 72±8 | 100 |
| Nguyen (2018) | Pharmacists’ training to improve inhaler technique of patients with COPD in vietnam | Vietnam | 211 | 66.6±8.2 | 84.4 |
| Norweg (2023) | Mind-Body Intervention for Dysfunctional Breathing in Chronic Obstructive Pulmonary Disease: feasibility Study and Lessons Learned | USA | 31 | 72.42±9.54 | 41.9 |
| O’Neill (2018) | Clinician-Facilitated Physical Activity Intervention Versus Pulmonary Rehabilitation for Improving Physical Activity in COPD: A Feasibility Study | UK | 49 | 64.4±8.6 | 49 |
| Park (2020) | Evaluating the effect of a smartphone app-based self-management program for people with COPD: A randomized controlled trial | Korea | 44 | 67.88±10.49 | 78.6 |
| Robinson (2021) | A randomised trial of a web-based physical activity self-management intervention in COPD | USA | 153 | 69.8±7.3 | 93 |
| Rutkowski (2021) | Evaluation of the Efficacy of Immersive Virtual Reality Therapy as a Method Supporting Pulmonary Rehabilitation: A Randomized Controlled Trial | Poland | 50 | 66±7.86 | 18 |
| Rutkowski (2020) | Virtual Reality Rehabilitation in Patients with Chronic Obstructive Pulmonary Disease: A Randomized Controlled Trial | Poland | 120 | 60.99 | 42.7 |
| Rutkowski (2019) | Effect of Virtual Reality‐Based Rehabilitation on Physical Fitness in Patients with Chronic Obstructive Pulmonary Disease | Poland | 68 | 61.3±3.7 | 51.5 |
| Simmich (2021) | A Co-Designed Active Video Game for Physical Activity Promotion in People with Chronic Obstructive Pulmonary Disease: Pilot Trial | Australia | 25 | 67.5±6.8 | 39 |
| Simmons (1996) | Trends in compliance with bronchodilator inhaler use between follow-up visits in a clinical trial | USA | 241 | 49.2 | Not reported |
| Song (2014) | Effectiveness of a brief self-care support intervention for pulmonary rehabilitation among the elderly patients with chronic obstructive pulmonary disease in Korea | Korea | 40 | 67.4±9.0 | 65 |
| Sutanto (2019) | Videogame assisted exercise training in patients with chronic obstructive pulmonary disease: A preliminary study | Indonesia | 23 | 65.35±6.10 | 95 |
| Tabak (2014) | A telerehabilitation intervention for patients with Chronic Obstructive Pulmonary Disease: a randomized controlled pilot trial | Netherlands | 34 | 66.6±7.4 | 65 |
| Tashkin (1991) | A nebulizer chronolog to monitor compliance with inhaler use | USA | 197 | 35-59 | Not reported |
| Vayisoglu (2019) | The health action process approach-based program's effects on influenza vaccination behavior | Turkey | 90 | 61.10±8.49 | 61 |
| Wewel (2008) | Intervention by phone calls raises domiciliary activity and exercise capacity in patients with severe COPD | Germany | 21 | 65±9 | 81 |
| Woo (2006) | A community model of group therapy for the older patients with chronic obstructive pulmonary disease: A pilot study | China | 44 | 74.2±6.5 | 84 |
| Wootton (2018) | Effect on health-related quality of life of ongoing feedback during a 12-month maintenance walking programme in patients with COPD: a randomized controlled trial | Australia | 95 | 69±8 | 58 |
| Yao (2020) | Application of peer support models in respiratory rehabilitation of patients with chronic obstructive pulmonary disease | China | 128 | 63.34±8.14 | 56 |
| Yao (2021) | Benefits Conferred by Peer-Support Nursing Intervention to Pulmonary Function and Quality of Life in Nonsmoking Patients with COPD | China | 100 | 45.81±15.10 | 55 |

# Supplementary Table 3 Intervention characteristics of included study

| First author (year) | Delivery Mode | Multi-component(M)/Single component(S) | Nudge element | Outcome measured | Description of intervention | Length of intervention | Description of control |
| --- | --- | --- | --- | --- | --- | --- | --- |
| Woo (2006) | Group session | M | Social influence | Exercise capacity, QoL (6MWD, SGRQ) | 8–10 subjects were grouped together and participated in a 12-week pulmonary rehabilitation programme. In the last session, the education session was replaced by a peer group sharing and discussion. This session allowed participants to share their experience when coping with the disease in daily living. | 12 weeks | NA |
| Yao (2020) | Mobile application | S | Social influence | QoL, Self-efficacy (QLQ-C30, ES-CA) | The peer support team provided relevant disease information to patients through telephone, WeChat and other platforms, and took respiratory rehabilitation training with patients at least once a day during hospitalization. | 3 months | Routine nursing plus respiratory rehabilitation training. |
| Yao (2021) | Mobile application | S | Social influence | Exercise capacity, Self-efficacy (6MWD, Health promotion self-care scale) | The patients received the health education training in the form of group interaction and by means of situational dialogue. The peers were assigned as group leaders to demonstrate the pulmonary function exercises and share disease rehabilitation knowledge. The patients were requested to become a member of peer-support WeChat group and participated in the group communication at 8 pm every Sunday. In the WeChat group, the disease rehabilitation knowledge and psychological counselling were shared by the nurses and peers, and the activity lasted for 1 h at least. | 3 months | Routine nursing intervention without the peers’ participation. |
| Burkow (2018) | Mobile application | M | Social influence | PA (The average number of physical activity sessions) | The participants exercised together in time but not at the same location via virtual group. The virtual group members see each other’s activity status. | 6 weeks | NA |
| Berry (2010) | Group session | M | Social influence | PA, Exercise capacity, QoL (PA levels, 6MWD, CRQ) | In the LAP intervention, the principles of group dynamics were used to systematically develop group formation and identity, create the group's common motivational base (i.e., independent physical activity), and establish exercise and adherence expectancies in members that were of consequence to the group. | 12 months | The control group received center-based exercise therapy and bimonthly education classes. |
| Sutanto (2019) | Wii console | S | Gamification | Exercise capacity, QoL, Clinical symptom (6MWD, SGRQ, MRC) | The experimental group (EG) received a hospital-based outpatient exercise training program and a Wii Fit videogame program. | 6 weeks | Exercise training program only. |
| Simmich (2021) | Mobile application | S | Gamification | PA (daily steps) | The patients took part in the co-design progress. In this game, the players completed upper body and lower body physical activities commonly used in the physical rehabilitation of people with COPD. The game features 2 parallel game modes, which can be used together or separately. | 3 weeks | The individuals did not take part in the co-design process, who received only the Fitbit activity tracker and the Fitbit app. |
| Rutkowski (2021) | VR device | S | Gamification | Exercise capacity, Pulmonary function (6MWD, FEV1%) | The VR-group performed 10 sessions of immersive VR-therapy and traditional PR programme. | 2 weeks | The control group performed 10 sessions of Schultz autogenic training and traditional PR programme. |
| Rutkowski (2020) | Xbox 360 console | S | Gamification | Exercise capacity (6MWD) | Patients in the ET+VR group participated in a TPR, as well as both endurance exercise training and VR sessions using the Kinect® system. Patients in VR group participated in a TPR and VR sessions. | 2 weeks | The patients in the ET group participated in a TPR program and endurance exercise training sessions. |
| Rutkowski (2019) | Xbox 360 console | S | Gamification | Exercise capacity (6MWD) | Patients participated in standard pulmonary rehabilitation, additionally taking part in daily training using the Kinect system. | 14 days | Standard pulmonary rehabilitation program. |
| Jung (2020) | VR headset | M | Gamification | QoL (CRQ) | PR in VR was designed to enable patients to perform various exercises in their homes as displayed in the VR environment and comprised 8 separate modules. | 8 weeks | NA |
| Colombo (2023) | Web based | S | Gamification | Exercise capacity (6MWD) | The VE represents a park with graphical and audio elements typical of a natural environment (e.g., flowers, trees, birds). The user navigates the VE in first-person perspective through a virtual bicycle that moves along a predefined path. | Around ten days | NA |
| Chau (2012) | Reminder device | S | Reminder | QoL, Pulmonary function (CRQ, FEV1(% predicted)) | Participants received the device kit, which had personalized reminders to take medication and perform breathing exercises to improve self-management. | 2 months | Education on self-care and symptom management techniques. |
| Song (2014) | Written material | M | Reminder | Exercise capacity, QoL, Medication adherence (6MWD, SGRQ, Medication adherence scale) | The written instruction, plus illustrations, was given to the participants as a reminder for instructional support and practice at home. | 2 months | Usual care, consisting of an education on COPD management, proven benefits of exercise, and maintaining daily activities. |
| Morfaw (2023) | Mobile application | S | Reminder | Medication adherence (MMAS-8) | The design of Medisafe makes it possible for individual patients to receive personalized messages reminding them about each of their medication that they are supposed to take including the expected side effects, and any possible drug interaction warnings. | 8 weeks | NA |
| Nguyen (2018) | Label | M | Reminder | Inhaler technique (Inhaler technique score) | If the patients made any mistake, one additional reminder label that included a summary of the steps was stuck onto their inhaler device(s) with a larger font size and patients were asked to confirm that they could read the label clearly. | 12 months | NA |
| Vayisoglu (2019) | Card | M | Reminder | Influenza vaccination, Self-efficacy (Influenza vaccination rate, Self-efficacy scale) | The vaccination card was developed to remind patients of the plan and support the self-efficacy of action that covered information about when and where to get an influenza vaccination. Telephone calls were intended to remind the time of vaccination and the plan. | 15 days | Routine influenza vaccination recommendations from health care personnel. |
| Criner (2021) | Mobile application | S | Reminder | Medication adherence, QoL (the mean number of adherent sets of puffs/day for 6 months, CCQ) | The intervention group received their established therapy and a BreatheMate medication monitoring device that provided twice-daily (once in the morning and once in the evening) auditory and visual reminders (beeps and flashes) via the BreatheMate device and a supportive application on the smartphone device. | 6 months | The control group received their established therapy and the BreatheMate medication monitoring device without any reminders. |
| Wewel (2008) | Pedometer | M | Feedback | Exercise capacity, QoL, PA (6MWD, SGRQ, Activity per hour of monitoring) | Patients wore an electronic actograph at the right side of their waist. Side by side with the actograph patients wore a pedometer, the readings of which were recorded in the diary. | 2 weeks | NA |
| Tashkin (1991) | Chronolog | S | Feedback | Medication adherence (The percent of patients actually used the inhaler two or more times daily according to the chronolog record) | The feedback group were informed of the function of the chronolog and were given feedback of the information derived from the chronolog in an attempt to enhance compliance. | 4 months | The uninformed group received the Nebulizer Chronolog but did not know that the chronolog was actually recording the exact time and date of each actuation. |
| Moy (2012) | Pedometer | M | Feedback | PA, QoL, Clinical symptom (Daily steps, SF-36, mMRC) | The pedometer, with on-instrument data presentation, provided subjects with continuous step-count feedback. | 3 months | NA |
| Robinson (2021) | Pedometer | M | Feedback | PA, Exercise capacity, QoL, Clinical symptom (Daily steps, 6MWD, SGRQ, mMRC) | Participants were asked to wear the pedometer every day, except while sleeping or showering/bathing, and to upload their step-count data to the study website as often as they wish, but at least weekly. An automated algorithm, developed by the investigators, computed gradually incrementing, individualized step-count goals based on data on website. | 6 months | Participants in control groups received verbal encouragement to increase daily physical activity and an education booklet that contained COPD self-management information. |
| Armstrong (2021) | Pedometer | M | Feedback | PA, Exercise capacity, QoL, Clinical symptom (Daily steps, 6MWD, CCQ, CAT) | Behavioral modification interventions comprised motivational interviews, monitoring and feedback using a pedometer and goal setting. | 8 weeks | The pulmonary rehabilitation programme alone. |
| O’Neill (2018) | Pedometer | S | Feedback | PA, Exercise capacity, QoL, Clinical symptom (Daily steps, Incremental Shuttle Walk Test, EQ5D, CAT) | Participants were provided with a pedometer and hence they could record and see their daily step count during the PAI. Each week participants set a step goal based on their previous weeks step count, as well as the results of a self-efficacy walk. | 12 weeks | Pulmonary rehabilitation. |
| Giardino (2004) | Physiologic Monitoring System, oximeter | S | Feedback | Exercise capacity, QoL, Self-efficacy, Pulmonary function (6MWD, SGRQ, COPD Self-efficacy scale, FEV1(% predicted)) | The patients participated in 5 weekly sessions of heart rate variability biofeedback and 4 weekly sessions of walking practice with oximetry feedback, with instructions for daily home practice. | 10 weeks | NA |
| Wootton (2018) | Pedometer | S | Feedback | Exercise capacity, QoL (6MWD, SGRQ) | After the 2-month intervention of a supervised, walking training programme, IG received telephone calls, biofeedback provided via a pedometer and progressive goal setting. | 12 months | The 2-month supervised, walking training programme. |
| Kawagoshi (2015) | Pedometer | S | Feedback | PA, Exercise capacity, QoL, Clinical symptom (The time spent walking, 6MWD, CRQ, MRC) | The subjects in the PR + P group completed the monitoring using a pedometer and received monthly feedback about their pedometer use by PR staff for 1 year in addition to the other aspects of the program described above for the PR group. | 12 months | Pulmonary rehabilitation. |
| Hospes (2009) | Pedometer | M | Feedback | PA, Exercise capacity, QoL, Self-efficacy (Daily steps, 6MWD, SGRQ, LIVAS scale) | A pedometer, worn all day during the intervention period, was used to monitor and support motivation to participate. | 12 weeks | Usual care. |
| Park (2020) | Pedometer | M | Feedback | PA, Exercise capacity, QoL, Self-efficacy, Clinical symptom (Daily steps, 6MWD, SF-36, SEMCD, UCSD-SOBQ) | Participants received a smartphone app-based self-management program, which included education, exercises, self-monitoring of symptoms and exercise, and social support. The patients were also asked to use a pedometer to increase their physical activity. They were asked to record the type and duration of exercise and step count from their pedometer in the smartphone app's exercise directory, whenever they exercised. | 6 months | Participants received sessions about education, exercises, self-monitoring, and social support. |
| Cruz (2014) | Activity monitors | M | Feedback | PA, Exercise capacity (Daily steps, 6MWD) | Daily PA was measured using activity monitors on weeks 1, 7, and 12, and feedback was given in the following weeks on the number of steps, time spent in sedentary, light, and moderate-to-vigorous intensity activities, and time spent standing, sitting, and lying. | 12 weeks | NA |
| Geidl (2021) | Pedometer | S | Feedback | PA, QoL, Clinical symptom (Daily steps, SGRQ, CAT) | In addition to the standard rehabilitation program, the intervention group received a pedometer-based PA promotion intervention. | 6 months | In addition to the 3-week inpatient rehabilitation, the control group received a revision of PA-related patient education of an equal duration with no pedometer. |
| Kohlbrenner (2020) | Pedometer | M | Feedback | PA, Exercise capacity, Clinical symptom (Daily steps, 1MSTS, CAT) | The intervention group received PA counselling and pedometer-based feedback in addition to usual care. The PA counselling program ended after three months while pedometers were provided until the end of the study at 12 months. | 12 months | Usual care, i.e., regular visits at their respiratory physician. |
| Arbillaga- Etx­­­arri (2018) | Pedometer | M | Feedback | PA, Exercise capacity, QoL, Clinical symptom (Daily steps, 6MWD, CCQ, CAT) | Urban Training consisted of a baseline motivational interview, advice to walk on urban trails designed for COPD patients in outdoor public spaces and other optional components for feedback, motivation, information and support (pedometer, calendar, physical activity brochure, website, phone text messages, walking groups and a phone number). | 12 months | Usual standardized pharmacological and/or non-pharmacological treatment for COPD. |
| Norweg (2023) | Capnograph | M | Feedback | Exercise capacity, QoL (6MWD, SGRQ) | Multi-component CART consisted of six, 1-h weekly sessions of slow breathing and mindfulness exercises, ETCO2 biofeedback, motivational counselling, and a home program. | 10 weeks | The center-based pulmonary rehabilitation program. |
| Tabak (2014) | Mobile application | S | Feedback | PA, QoL, Clinical symptom (Daily steps, CCQ, MRC) | The telerehabilitation application consists of an activity coach (3D-accelerometer with smartphone) for ambulant activity registration and real-time feedback, complemented by a web portal with a symptom diary for self-treatment of exacerbations. | 3 weeks | Usual care. |
| Mendoza (2015) | Pedometer | S | Feedback | PA, Exercise capacity, QoL, Clinical symptom (Daily steps, 6MWD, SGRQ, CAT) | Participants were encouraged to be more active by using the pedometer to measure the number of steps walked daily and to record this in the diary provided, together with any information related to their clinical condition. | 3 months | The standard programme of physical activity encouragement. |
| Jolly (2018) | Pedometer | M | Feedback | PA, QoL, Medication adherence, Self-efficacy, Smoking cessation, Clinical symptom (Total MET minutes/week, SGRQ, Medication adherence score, Stanford self efficacy scale, Smoking cessation rate, MRC) | The intervention consisted of telephone health coaching delivered by a nurse with supporting written documents, a pedometer, and a self monitoring diary. | 6 months with 12 months follow up | The usual care group received a standard information leaflet about self management of COPD. |
| Altenburg (2015) | Pedometer | M | Feedback | PA, Exercise capacity, QoL (Daily steps, 6MWD, CCQ, CRQ) | The individual counselling was predominantly based on principles of goal-setting and implementation of goals and motivational interviewing techniques were used. Patients wore a pedometer all day during the intervention period, which was used for feedback and motivation. | 3 months with 15 months follow up | The usual care group received care appropriate to their health status. |
| Collins (2019) | Metronome | S | Feedback | QoL (CRQ) | The intervention consisted of exercise­training plus breathing-retraining using acoustic feedback. | 12 weeks | Exercise-training alone |
| de Blok (2006) | Pedometer | M | Feedback | PA, Exercise capacity, QoL, Self-efficacy (Daily steps, 2MWT, SGRQ, LIVAS) | The experimental group followed a lifestyle physical activity counselling program with feedback of a pedometer next to the regular pulmonary rehabilitation program. The pedometer was used as a motivational and feedback tool. | 9 weeks | Pulmonary rehabilitation. |
| Esteve (1996) | Lung function test device | S | Feedback | Pulmonary function  (FEV1(% predicted)) | Patients in the breathing pattern training group provided with visual feedback adjusted the shape of each breath (i.e., the plot of amplitude as a function of time) to a long and deep breath drawn from their own ventilatory repertoire. | 4 weeks | Standard physiotherapy. |
| Simmons (1996) | Chronolog | S | Feedback | Medication adherence (mean sets per day) | Participants were aware of the NC's exact monitoring function. In the feedback group, the readings of actuation dates and times from the NC were used for feedback during the first and tenth weeks following their smoking cessation group's "quit" date and at each 4-month follow-up in an effort to enhance compliance with the prescribed medication regimen. | 24 months | Participants were not informed of the date- and time-recording capabilities of the attached NC, although they were aware that the NC would be monitoring the total amount of medication used. |
| Cruz (2016) | Pedometer | M | Feedback | PA, Exercise capacity, QoL, Self-efficacy (Daily steps, 6MWD, SGRQ, Self-Efficacy Scale) | The PA-focused behavioral intervention consisted of the health contract technique and objective feedback provided by pedometers based on social cognitive theory. | 6 months | Pulmonary rehabilitation. |

# Supplementary Table 4 EPOC risk of assessment for studies

| First Author (year) | Random sequence generation | | Allocation concealment | | Baseline outcome measurements similar | | Baseline characteristics similar | Incomplete outcome data | Knowledge of allocated interventions adequately prevented | | Protection against contamination | | Selective outcome reporting | | Other risks of bias |  |  |
| --- | --- | --- | --- | --- | --- | --- | --- | --- | --- | --- | --- | --- | --- | --- | --- | --- | --- |
|  | **Studies with a separate control group** | | | | | | | | | | | | | | | | |
| Altenburg (2015) | L | | U | | U | | U | L | L | | L | | L | | L |  |  |
| Arbillaga-Etxarri (2018) | U | | U | | L | | L | H | L | | L | | L | | L |  |  |
| Armstrong (2021) | U | | U | | U | | L | L | L | | L | | L | | L |  |  |
| Berry (2010) | L | | L | | U | | L | L | L | | L | | L | | L |  |  |
| Chau (2012) | L | | U | | L | | U | L | H | | L | | L | | L |  |  |
| Collins (2019) | U | | U | | U | | L | L | L | | L | | L | | L |  |  |
| Criner (2021) | L | | L | | H | | L | H | L | | L | | L | | L |  |  |
| Cruz (2016) | L | | L | | L | | L | L | L | | L | | L | | L |  |  |
| de Blok (2006) | U | | U | | U | | L | L | L | | L | | L | | L |  |  |
| Esteve (1996) | U | | U | | L | | H | L | L | | L | | L | | L |  |  |
| Geidl (2021) | L | | L | | L | | U | L | L | | L | | L | | L |  |  |
| Hospes (2009) | U | | U | | L | | L | L | L | | L | | L | | L |  |  |
| Jolly (2018) | L | | L | | L | | L | H | H | | L | | L | | L |  |  |
| Kawagoshi (2015) | U | | U | | L | | L | L | L | | L | | L | | L |  |  |
| Kohlbrenner (2020) | L | | L | | H | | L | L | L | | L | | L | | L |  |  |
| Mendoza (2015) | L | | U | | L | | L | L | L | | L | | L | | L |  |  |
| Norweg (2023) | L | | L | | U | | U | L | L | | L | | L | | L |  |  |
| O’Neill (2018) | L | | L | | U | | U | H | L | | L | | L | | L |  |  |
| Park (2020) | L | | U | | L | | L | L | H | | L | | L | | L |  |  |
| Robinson (2021) | L | | U | | L | | L | L | L | | L | | L | | L |  |  |
| Rutkowski (2019) | L | | L | | L | | L | L | L | | L | | L | | L |  |  |
| Rutkowski (2020) | L | | L | | L | | L | L | L | | L | | L | | L |  |  |
| Rutkowski (2021) | L | | L | | H | | L | L | L | | L | | L | | L |  |  |
| Simmich (2021) | H | | H | | U | | L | L | L | | L | | L | | L |  |  |
| Simmons (1996) | L | | U | | H | | H | L | L | | L | | L | | L |  |  |
| Song (2014) | H | | U | | L | | L | L | U | | L | | L | | L |  |  |
| Sutanto (2019) | L | | U | | U | | L | L | L | | L | | L | | L |  |  |
| Tabak (2014) | L | | U | | U | | L | U | L | | L | | L | | L |  |  |
| Tashkin (1991) | H | | U | | H | | H | L | L | | L | | L | | L |  |  |
| Vayisoglu (2019) | L | | U | | U | | L | L | U | | L | | L | | L |  |  |
| Wootton (2018) | L | | L | | U | | L | H | L | | L | | L | | L |  |  |
| Yao (2020) | U | | U | | L | | L | L | U | | L | | L | | L |  |  |
| Yao (2021) | H | | U | | L | | L | L | U | | L | | L | | L |  |  |
|  | | **Interrupted time series studies** | | | | | | | | | | | | | |  |  |
| Author (year) | | **Intervention independent of other changes** | | **Shape of the intervention effect pre-specified** | | **Intervention unlikely to affect data collection** | | **Knowledge of the allocated interventions adequately prevented during the study** | | **Incomplete outcome data adequately** | | **Selective outcome reporting** | | **Other risks of bias** | | |  |
| Burkow (2018) | | L | | L | | L | | L | | L | | L | | L | | |  |
| Colombo (2023) | | L | | L | | L | | L | | U | | L | | L | | |  |
| Cruz (2014) | | L | | L | | L | | L | | L | | L | | L | | |  |
| Giardino (2004) | | L | | L | | L | | L | | L | | L | | L | | |  |
| Jung (2020) | | L | | L | | L | | U | | L | | L | | L | | |  |
| Morfaw (2023) | | L | | L | | L | | L | | L | | L | | L | | |  |
| Moy (2012) | | L | | L | | L | | L | | L | | L | | L | | |  |
| Nguyen (2018) | | L | | L | | L | | L | | L | | L | | L | | |  |
| Wewel (2008) | | L | | L | | L | | U | | L | | L | | L | | |  |
| Woo (2006) | | L | | L | | L | | U | | U | | L | | L | | |  |

**L: low risk of bias H: high risk of bias U: unclear risk of bias**
